# Evaluating LingualAI: a prospective validation of AI-based real-time translation against certified human interpreters

**DOI:** 10.1038/s44401-026-00080-5

**Published:** 2026-05-12

**Authors:** Uday P. Singh, Carlos A. Jaimes Garcia, Gabriel M. Aisenberg, Javier Barreda Garcia, Jessica A. Hernandez-Chilatra, Cecilia Wang, Dalilah Reyes de Jesus, Eileen Whalen, Veronica Santos Canellas, Amanda R. Falk Vargas, Brian O. Rodriguez Echevarria, Martin J. Citardi, Babatope O. Fatuyi, Xiaoqian Jiang

**Affiliations:** 1 McWilliams School of Biomedical Informatics, University of Texas Health Science Center at Houston, Houston, TX USA; 2 McGovern Medical School, University of Texas Health Science Center at Houston, Houston, TX USA

**Keywords:** Health care, Medical research

## Abstract

Limited English proficiency affects over 25 million people in the United States and is associated with disparities in healthcare access, safety, and outcomes. We conducted a prospective, within-subject, simulation-based comparison to evaluate whether an in-house AI application (LingualAI) achieves non-inferior translation quality versus certified medical interpreters in English–Spanish otorhinolaryngology encounters. Standardized clinician–patient scripts were translated by LingualAI and by certified interpreters, and bilingual clinicians rated anonymized audio across multidomain quality measures. Using a prespecified non-inferiority margin of 0.30 points (Human − AI) on 5-point scales, LingualAI met non-inferiority for 2 of 3 primary factors (terminology accuracy Δ = 0.07; adequacy of meaning Δ = 0.13) but not clarity (Δ = 0.50). It met non-inferiority for 1 secondary factor (completeness Δ = 0.14), while grammar (Δ = 0.21; upper 95% CI = 0.34), vocabulary (Δ = 0.18; upper 95% CI = 0.32), and cultural appropriateness (Δ = 0.39) exceeded the margin. No voice-related factors met non-inferiority (fluency Δ = 1.13; prosody Δ = 0.59; pacing Δ = 0.40), and conclusive ratings favored interpreters (overall quality Δ = 0.58; clinical confidence Δ = 0.61). These findings suggest LingualAI preserves core clinical meaning and terminology but remains limited by speech naturalness and delivery, supporting use as an adjunct when interpreter access is constrained and favoring interpreter-in-the-loop deployment for higher-stakes communication.

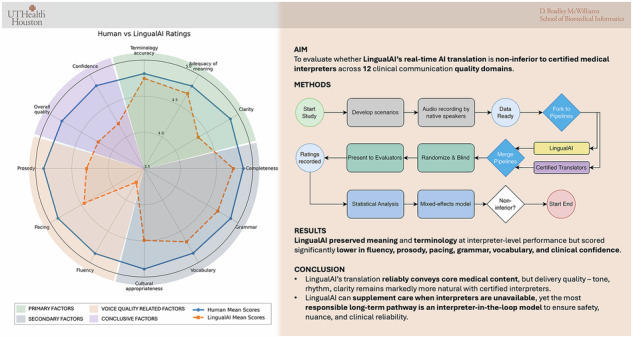

## Introduction

Effective communication is fundamental to safe and equitable healthcare. In countries with substantial linguistic diversity, such as the United States, a growing proportion of clinical encounters involve patients with limited English proficiency (LEP), estimated at approximately 25 million people, or about 8% of the population aged 5 years and older who report speaking English less than “very well”^1,2^. LEP patients face barriers to understanding diagnoses, treatment instructions, and follow-up care, contributing to disparities in safety, quality, and health outcomes^3,4^. A recent systematic review found that when LEP patients receive language-concordant care, clinical outcomes are improved in most settings^1^. Although professional medical interpreters are the standard of care, access is often limited, particularly in primary care, rural settings, and time-sensitive encounters, leaving many patients without reliable language support^5,6^.

Recent advances in artificial intelligence (AI) have enabled speech- and language-model–based systems capable of real-time multilingual translation^7,8^. Several mobile or app-based tools are now available at the point of care; yet systematic evidence regarding their performance, accuracy, and clinical appropriateness remains scarce. In particular, little is known about how AI-based translations compare with certified human interpreters when assessed against clinical communication standards such as terminology accuracy, adequacy of meaning, cultural appropriateness, and speech fluency. Notably, in a recent study evaluating three commercially available applications for bidirectional clinician–patient dialogue, none were found to be suitable for safe two-way clinical communication when compared with professional interpreters^9^. This evidence gap complicates safe integration into health systems.

The LingualAI application was created to reduce language barriers in clinical settings through real-time voice translation. Developed at UTHealth Houston, its purpose is to help clinical team members and patients communicate seamlessly through a continuous flow of voice capture, transcription, translation, and synthesis. Its interface is designed to be intuitive: users can select patient and clinician languages, record conversations linked to each patient, and view interactive bubbles on screen with real-time transcription and translation. In addition, the app offers translation quality feedback, conversation history, secure device synchronization, and an offline mode that ensures reliability even in low-connectivity environments. However, its effectiveness relative to certified medical interpreters has not been formally evaluated.

We conducted a prospective, within-subject comparative simulation-based study using scripted bilingual dialogues to evaluate LingualAI’s performance in English–Spanish interactions in an outpatient otorhinolaryngology setting. We assess whether LingualAI’s audio translations are non-inferior to certified medical interpreters across multiple domains of translation quality, including terminology accuracy, adequacy of meaning, clarity/fluency, cultural appropriateness, and overall confidence for clinical use. By applying a structured validation framework, this work provides early, controlled evidence of LingualAI’s translation quality and feasibility, rather than definitive validation for unrestricted real-world clinical deployment.

## Results

Results are organized in three analytical layers. Differences are reported as Δ = Human − AI (positive values favor human), with a prespecified non-inferiority margin of 0.30 points on the 5-point scale. Analyses include both paired and mixed-effects models to account for evaluator clustering and confirm robustness. Findings are presented as follows: (i) evaluator participation and reliability, (ii) domain-level performance evaluated using non-inferiority testing and direction-specific contrasts, and (iii) secondary analyses, including preference patterns, robustness checks, and representative error types.

### Evaluator and data overview

Nine bilingual clinicians contributed ratings across three standardized scenarios comprising 18 clinician and 15 patient lines. Evaluator participation varied by scenario (8, 7, and 4 raters for Scenarios 1–3, respectively), resulting in an unbalanced rater–item panel. Participation declined across scenarios due to evaluator fatigue and the substantial time required, leading to fewer ratings in Scenario 3 (Table 1). Because raters were anonymous, inter-rater reliability was estimated using Krippendorff’s α, which accommodates missing data and unequal numbers of raters per item. Across all domains, α = 0.31, reflecting fair agreement. The modest inter-rater reliability reflects systematic differences in clinician perceptual thresholds, particularly for voice-related domains such as fluency, prosody, and clinical confidence. This variability was compounded by unbalanced evaluator participation across scenarios and the absence of formal rater calibration, as the study was intentionally designed to capture independent, first-impression clinical judgments rather than consensus scoring. Importantly, this heterogeneity reflects differences in scoring standards across clinicians rather than random measurement error and was mitigated analytically using mixed-effects models with random intercepts for dialogue line. Despite this variability, consistent directional differences between AI and human translations were observed across raters and domains, supporting cautious interpretation of the findings. These results reflect performance under standardized, simulation-based conditions and should be interpreted as controlled evidence of translation quality rather than direct measurement of real-world clinical encounter performance. Domain-specific Krippendorff’s α values are reported in Table 2. Fluency and prosody violated normality assumptions and were therefore analyzed using non-parametric tests.

**Table 1:** Evaluator participation and rating distribution across scenarios: Each scenario included 11 dialogue lines (6 clinicians, 5 patients)

Scenario	Number of evaluators	Clinician dialogue lines	Patient dialogue lines	AI translation ratings	Human interpreter ratings	Total ratings
Scenario 1	8	6	5	1056	1056	2112
Scenario 2	7	6	5	924	924	1848
Scenario 3	4	6	5	528	528	1056

Two AI and two human outputs per line were rated by bilingual clinicians, with totals reflecting combined evaluations for both translation arms.

**Table 2:** Domain-level rating distribution and inter-rater reliability: Mean scores, variability measures, and Krippendorff’s α are shown for each translation quality domain across all evaluators and scenarios

Domain category	Quality domain	Number of ratings	Mean ± SD	Median	Q1	Q3	IQR	Krippendorff’s α
Primary factors	Terminology accuracy	418	4.78 ± 0.66	5	5	5	0	0.41
Primary factors	Adequacy of meaning	418	4.76 ± 0.67	5	5	5	0	0.37
Primary factors	Clarity	418	4.62 ± 0.86	5	5	5	0	0.25
Secondary factors	Completeness	418	4.80 ± 0.68	5	5	5	0	0.26
Secondary factors	Grammar	418	4.78 ± 0.64	5	5	5	0	0.35
Secondary factors	Vocabulary	418	4.76 ± 0.63	5	5	5	0	0.3
Secondary factors	Cultural appropriateness	418	4.69 ± 0.84	5	5	5	0	0.25
Voice-related factors	Fluency	418	4.29 ± 1.20	5	4	5	1	0.31
Voice-related factors	Pacing	418	4.67 ± 0.75	5	5	5	0	0.24
Voice-related factors	Prosody	418	4.59 ± 0.86	5	5	5	0	0.26
Conclusive factors	Overall quality	418	4.52 ± 0.93	5	4	5	1	0.29
Conclusive factors	Clinical confidence	418	4.52 ± 0.96	5	4	5	1	0.29

Ratings used 5-point Likert scales (1 = poor, 5 = excellent).

Although evaluators were blinded to the translation source, it is possible that some raters inferred AI-generated outputs due to the use of a consistent synthetic voice and more mechanistic delivery. Such awareness or suspicion may have influenced ratings, particularly in voice-related domains (fluency, prosody, pacing), as well as overall quality and clinical confidence. Importantly, any such effect would be expected to bias results against AI performance rather than inflate it.

### Primary Analyses (fixed- and mixed effects)

Results from paired and mixed-effects models were largely consistent (Table 3). Adjusting for evaluator clustering did not alter the direction or significance of findings, confirming the robustness of observed differences between human and AI translations.

**Table 3:** Paired and mixed-effects comparison between human and AI translation ratings: Mean ratings, test statistics, and effect sizes are shown for each translation quality domain

Domain category	Quality domain	Human mean rating (*n* = 19)	AI mean rating (*n* = 19)	Statistical test	Test statistic	Two-sided *p*-value	Effect size	Mixed-effects adjusted mean difference (Human − AI)	95% CI (mixed-effects model)	Mixed-effects p-value
Primary factors	Terminology accuracy	4.81	4.75	Paired t-test	0.92	0.368	*d* = 0.21	0.07	−0.05 to 0.19	0.282
Primary factors	Adequacy of meaning	4.82	4.7	Paired t-test	2.46	0.024	*d* = 0.56	0.12	−0.00 to 0.24	0.05
Primary factors	Clarity	4.88	4.36	Wilcoxon (exact)	2	<0.001	*r* = 0.76	0.51	0.39 to 0.63	<1 × 10⁻¹⁶
Secondary factors	Completeness	4.87	4.73	Wilcoxon (exact)	10	0.022	*r* = 0.53	0.14	0.02 to 0.26	0.021
Secondary factors	Grammar	4.88	4.67	Wilcoxon (exact)	5	0.007	*r* = 0.62	0.21	0.09 to 0.32	<0.001
Secondary factors	Vocabulary	4.85	4.67	Wilcoxon (exact)	27	0.06	*r* = 0.43	0.18	0.07 to 0.29	0.001
Secondary factors	Cultural appropriateness	4.89	4.49	Wilcoxon (exact)	12	0.006	*r* = 0.63	0.4	0.27 to 0.52	<1 × 10⁻⁹
Voice-related factors	Fluency	4.86	3.72	Wilcoxon (exact)	3.5	<0.001	*r* = 0.96	1.14	0.97 to 1.31	<1 × 10⁻³⁹
Voice-related factors	Pacing	4.88	4.46	Wilcoxon (exact)	4.5	0.002	*r* = 0.72	0.42	0.31 to 0.53	<1 × 10⁻¹³
Voice-related factors	Prosody	4.89	4.3	Wilcoxon (exact)	4	<0.001	*r* = 0.76	0.59	0.47 to 0.72	<1 × 10⁻¹⁹
Conclusive factors	Overall quality	4.81	4.23	Wilcoxon (exact)	2.5	0.001	*r* = 0.75	0.58	0.44 to 0.72	<1 × 10⁻¹⁵
Conclusive factors	Clinical confidence	4.82	4.22	Wilcoxon (exact)	1.5	0.001	*r* = 0.74	0.61	0.47 to 0.75	<1 × 10⁻¹⁶

Paired t-tests or Wilcoxon tests were applied based on normality (Shapiro–Wilk). Mixed-effects models included random intercepts for item (line) to account for repeated ratings. Positive Δ values indicate higher human scores.

### Primary factors

Adequacy of meaning (human 4.82 vs. AI 4.70; 95% CI, 0.00–0.24; *p* = 0.050) and terminology accuracy (4.81 vs. 4.75; CI, −0.05–0.19; *p* = 0.282) remained statistically comparable across both models, indicating semantic equivalence between human and AI outputs. In contrast, clarity showed a consistent and large human advantage (4.88 vs. 4.36; *p* < 0.001; *r* = 0.76).

### Secondary factors

Human interpreters achieved higher scores in completeness (4.87 vs. 4.73; *p* = 0.022; 95% CI, 0.02–0.26), grammar/syntax (4.88 vs. 4.67; *p* = 0.007; CI, 0.09–0.32), vocabulary (4.85 vs. 4.67; *p* = 0.060; CI, 0.07–0.29), and cultural appropriateness (4.89 vs. 4.49; *p* < 0.001; CI, 0.27–0.52). Effect sizes ranged from moderate for vocabulary to large for grammar/syntax and cultural appropriateness.

### Voice-quality factors

The most pronounced differences were observed in fluency (4.86 vs. 3.72; *p* < 0.001; CI, 0.97–1.31), prosody (4.89 vs. 4.30; *p* < 0.001; CI, 0.47–0.72), and pacing (4.88 vs. 4.46; *p* = 0.002; CI, 0.31–0.53), all demonstrating large, statistically robust advantages for human interpreters.

### Conclusive factors

Overall quality (4.81 vs. 4.23; *p* < 0.001; CI, 0.44–0.72) and clinical confidence (4.82 vs. 4.22; *p* < 0.001; CI, 0.47–0.75) were also significantly higher for human translations.

### Direction-specific contrasts

Stratified analyses confirmed these trends (Supplementary Material, SM4). For clinician statements (English → Spanish), human interpreters outperformed the AI system in clarity, fluency, prosody, pacing, overall quality, and clinical confidence, while terminology and adequacy of meaning were equivalent. For patient statements (Spanish → English), human interpreters again scored higher across delivery-related and linguistic-mechanics domains (grammar, vocabulary, prosody), whereas accuracy and meaning remained non-inferior.

### Non-inferiority testing

**Fig. 1:**
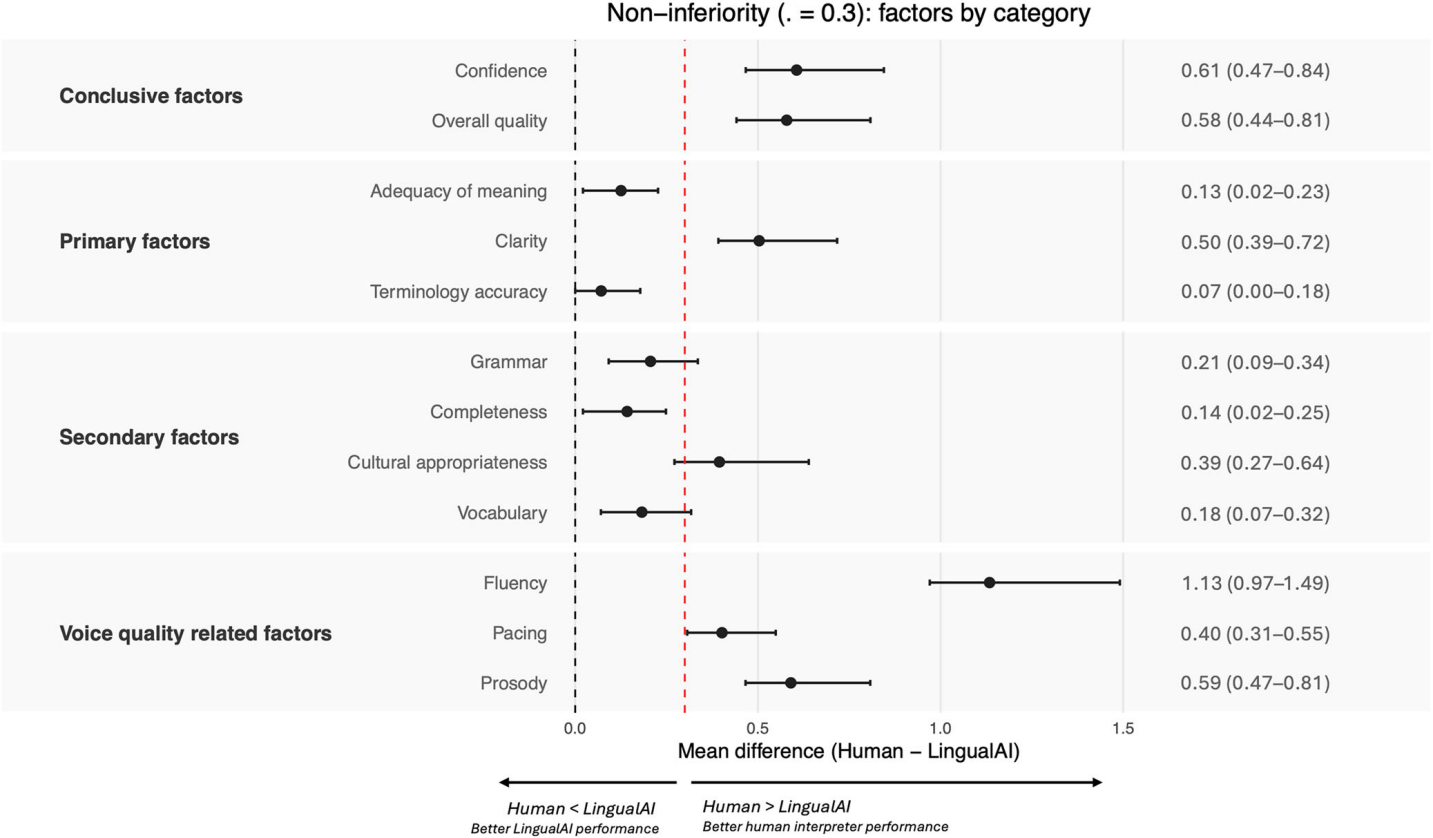
Non-inferiority analysis of AI versus human translation across quality domains. Mean differences (Human − LingualAI) with 95% confidence intervals are shown for 12 translation quality domains, grouped by category. The vertical dashed line at Δ = 0 indicates no difference, and the red dashed line marks the prespecified non-inferiority margin (Δ = 0.30). Domains with confidence intervals entirely to the left of the non-inferiority margin meet criteria for non-inferior AI performance. LingualAI met non-inferiority for terminology accuracy and adequacy of meaning (primary factors) and for completeness (secondary factor). All voice-related domains (fluency, prosody, pacing) and conclusive domains (overall quality, clinical confidence) exceeded the non-inferiority margin, indicating superior performance by certified medical interpreters in these areas.

A prespecified non-inferiority margin of 0.30 points on the 5-point scale was applied (Fig. 1). Only a subset of domains met this threshold, indicating that while LingualAI preserved semantic accuracy, delivery-related aspects remained inferior to human interpreters.

#### Primary factors

Terminology accuracy (mean difference 0.07; 95% CI, 0.18) and adequacy of meaning (0.13; CI, 0.23) satisfied the non-inferiority criterion, confirming comparable semantic performance. Clarity exceeded the margin (0.50; CI, 0.72), reflecting a perceptible decline in intelligibility relative to human translation.

#### Secondary factors

Completeness (0.14; CI, 0.25) met the criterion, but vocabulary (0.18; CI, 0.32), grammar (0.21; CI, 0.34), and cultural appropriateness (0.39; CI, 0.64) exceeded the margin, suggesting subtle but consistent linguistic differences.

#### Voice-quality factors

None met the non-inferiority threshold, differences were largest for fluency (1.13; CI, 1.49), followed by prosody (0.59; CI, 0.81) and pacing (0.40; CI, 0.55), indicating that human interpreters retained a clear advantage in speech naturalness and rhythm.

#### Conclusive factors

Both overall quality (0.58; CI, 0.81) and clinical confidence (0.61; CI, 0.84) exceeded the margin, underscoring the evaluators’ preference and higher perceived reliability for human translations. The mean differences and 95% confidence intervals for all domains are visualized in Table 4 and Fig. 1, plotted against the prespecified non-inferiority boundary.

**Table 4:** Non-inferiority testing of AI versus human translation across quality domains: Mean differences (Human − AI), 95% confidence intervals, and non-inferiority outcomes are shown for each domain

Domain category	Quality domain	n (paired ratings)	Mean difference (Human − AI)	Upper bound of 95% CI	Non-inferiority margin (Δ)	Non-inferior	Non-inferiority p-value (one-sided)
Primary factors	Terminology accuracy	38	0.071053	0.178035	0.3	TRUE	0.00045
Primary factors	Adequacy of meaning	38	0.125439	0.226835	0.3	TRUE	0.003087
Primary factors	Clarity	38	0.503509	0.717044	0.3	FALSE	0.941819
Secondary factors	Completeness	38	0.142105	0.248403	0.3	TRUE	0.008368
Secondary factors	Grammar	38	0.20614	0.335727	0.3	FALSE	0.114725
Secondary factors	Vocabulary	38	0.182456	0.317448	0.3	FALSE	0.075138
Secondary factors	Cultural appropriateness	38	0.394737	0.639347	0.3	FALSE	0.741233
Voice-related factors	Fluency	38	1.134211	1.490901	0.3	FALSE	0.999829
Voice-related factors	Pacing	38	0.401754	0.548884	0.3	FALSE	0.874619
Voice-related factors	Prosody	38	0.590351	0.807625	0.3	FALSE	0.984914
Conclusive factors	Overall quality	38	0.578947	0.808039	0.3	FALSE	0.976467
Conclusive factors	Clinical confidence	38	0.60614	0.844966	0.3	FALSE	0.981447

A prespecified non-inferiority margin of 0.30 points on the 5-point scale was applied. LingualAI was considered non-inferior when the upper bound of the 95% CI was below this margin.

When a stricter non-inferiority margin of 0.20 was applied, limited to Scenarios 1–2 due to smaller sample size in Scenario 3, terminology accuracy remained non-inferior while all other domains exceeded the margin. Detailed results are provided in Supplementary Material (SM5).

### Preference analysis

Pairwise preference testing compared individual evaluator choices between human and AI-generated audio clips for each domain (Table 5). Overall, preference patterns closely mirrored the quantitative scoring results: evaluators perceived both systems as equivalent for meaning and terminology but favored human interpreters for delivery quality and naturalness.

**Table 5:** Pairwise preference analysis between human and AI translations: Evaluator preferences are summarized as tie rates, human win rates, and AI win rates for each domain, with net bias representing the difference between human and AI win proportions

Domain category	Quality domain	Tie rate	Human preferred (%)	AI preferred (%)	Net preference (Human − AI)
Primary factors	Terminology accuracy	0.66	17.5	16.7	0.01
Primary factors	Adequacy of meaning	0.65	21.9	13.2	0.09
Primary factors	Clarity	0.55	40.4	4.4	0.36
Secondary factors	Completeness	0.73	18.4	8.8	0.1
Secondary factors	Grammar	0.63	28.9	7.9	0.21
Secondary factors	Vocabulary	0.58	27.2	14.9	0.12
Secondary factors	Cultural appropriateness	0.61	30.7	7.9	0.23
Voice-related factors	Fluency	0.25	66.7	7.9	0.59
Voice-related factors	Pacing	0.51	43.9	5.3	0.39
Voice-related factors	Prosody	0.49	44.7	6.1	0.39
Conclusive factors	Overall quality	0.42	48.2	9.6	0.39
Conclusive factors	Clinical confidence	0.41	49.1	9.6	0.39

Higher net bias values indicate a stronger preference for human translations. Evaluators most frequently favored human outputs for delivery-related and conclusive factors, while meaning and terminology domains showed high tie rates reflecting perceived equivalence.

#### Primary factors

Terminology accuracy (tie rate, 66%) and adequacy of meaning (65%) showed high equivalence, with minimal net bias toward human translations. Clarity revealed a stronger human preference (40% human wins vs. 4% AI wins; net bias of 0.36).

#### Secondary factors

Completeness also demonstrated a high tie rate (73%), whereas grammar (29% vs. 8%; net bias of 0.21) and cultural appropriateness (31% vs. 8%; net bias of 0.23) favored human interpreters. Vocabulary showed a smaller but consistent human advantage (net bias of 0.12).

#### Voice-quality factors

Preferences were most pronounced in these domains. Fluency had the lowest tie rate (25%) and the highest human win rate (67%; net bias of 0.59), followed by pacing and prosody, both exhibiting substantial net biases (>0.38).

#### Conclusive factors

For overall quality and clinical confidence, human translations won in roughly half of all comparisons (~49%), with average net biases near 0.39. These findings emphasize that, while evaluators recognized comparable semantic accuracy, they consistently preferred the tone, rhythm, and expressiveness of human interpretations. Detailed tie rates for each domain can be found in Supplementary Material SM6.

### Error analysis

**Fig. 2:**
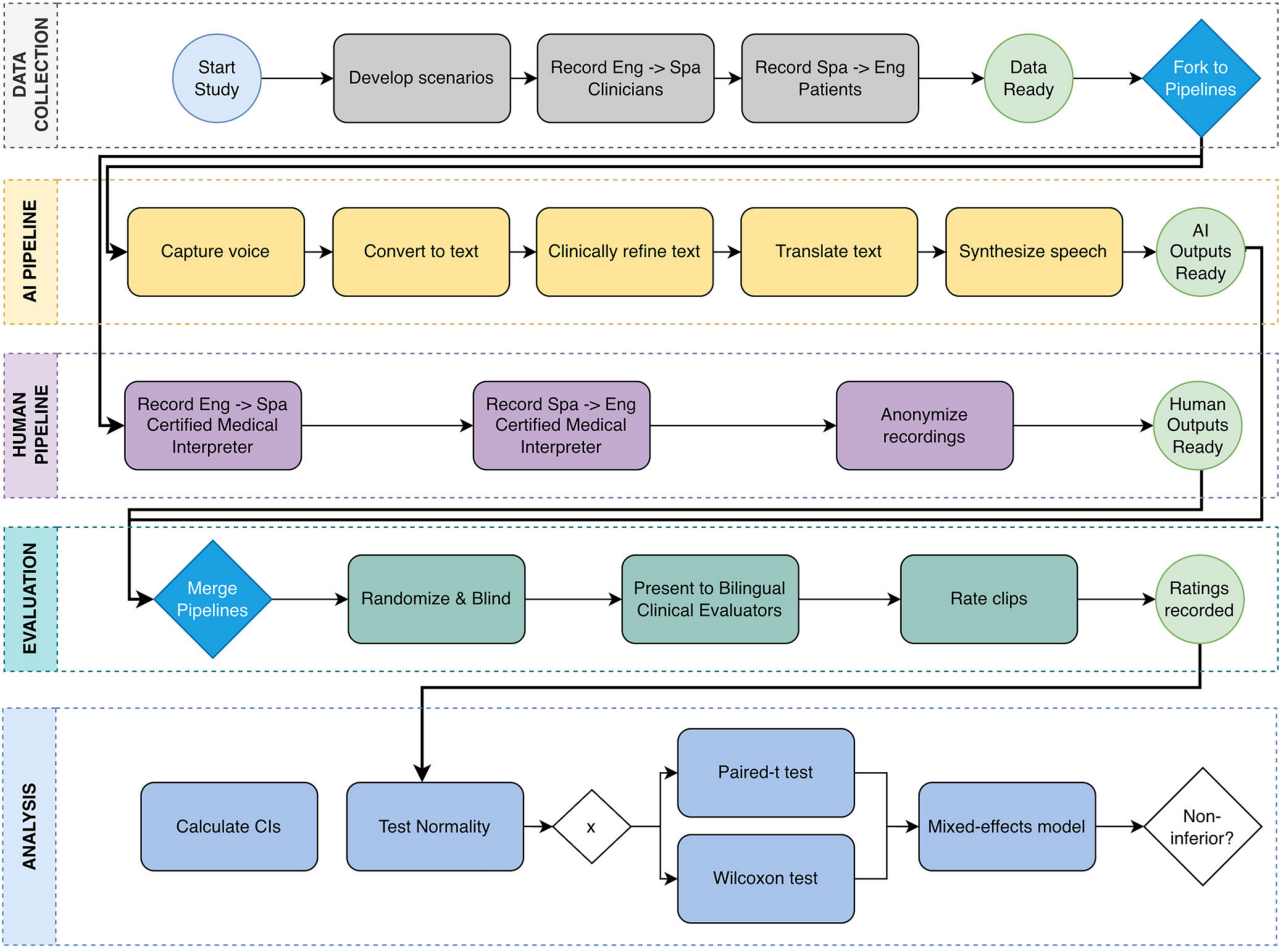
Study design and analytic workflow. The workflow illustrates the sequential stages of data collection, translation, evaluation, and analysis. Three standardized otorhinolaryngology scenarios were enacted by native English- and Spanish-speaking clinician–patient pairs and forked into two parallel translation pipelines. In the AI pipeline, speech was captured, converted to text, clinically refined, translated, and synthesized into speech. In the human pipeline, certified medical interpreters independently performed English→Spanish and Spanish→English translation, generating parallel recordings that were subsequently anonymized. Outputs from both pipelines were merged, randomized, and presented to blinded bilingual clinician evaluators for multidomain quality rating. Statistical analyses included paired tests and mixed-effects models with a prespecified non-inferiority margin of 0.30 points on 5-point Likert scales.

The present evaluation was conducted under controlled, simulation-based conditions; however, errors in AI-assisted translation may arise at multiple stages of the pipeline as illustrated in Fig. 2 when systems such as LingualAI are deployed in real-world clinical settings. Upstream errors may occur during voice capture and speech-to-text conversion due to background noise, accents, or incomplete utterances. Mid-pipeline errors may occur during clinical text refinement or translation, particularly when ambiguous terminology or context-dependent expressions are present. Downstream errors may occur during speech synthesis, leading to prosody, pacing, or emphasis that do not align with clinical intent. Failures at earlier stages can propagate, compounding their impact on the final output quality.

Within the controlled conditions of this study, a qualitative review of evaluator comments and audio outputs revealed that most residual errors arose from delivery rather than meaning. Specifically, deviations in fluency and prosody, such as monotone intonation, abrupt phrasing, and irregular pacing, occasionally disrupted conversational flow, even when lexical and semantic accuracy were preserved.

These patterns were observed across all scenarios. For example, certain clinician prompts (e.g., “Are you still doing the Budesonide irrigations?”) exhibited misplaced pauses or unnatural emphasis, while reassurance statements (e.g., “That’s expected. The swelling should go down over the next few weeks.”) were rendered with rigid or mechanical rhythm. Importantly, semantic fidelity remained high, indicating that LingualAI’s primary limitation in this evaluation lay in speech synthesis naturalness rather than translation accuracy.

Representative examples with linked audio clips are provided in Supplementary Material SM7, illustrating how subtle deviations in tone and pacing can alter perceived empathy and conversational realism. In deployed settings, LingualAI is designed to mitigate such risks by monitoring confidence signals and enabling escalation to certified medical interpreters when uncertainty, ambiguity, or high-stakes content is detected.

### Translation latency and cost

Based on system log analyses, LingualAI achieved an average end-to-end translation latency of 9.7 s per message, encompassing automatic speech recognition, neural translation, and speech synthesis. This turnaround time falls within the typical conversational pause window, allowing smooth, turn-based clinical dialogue without noticeable interruption.

The estimated operating cost for a 10-min bilingual conversation was approximately $0.03–$0.04 (USD), compared with $6.90–$10.60 for phone or video interpreter services, representing a cost reduction exceeding 99% per session. These results highlight LingualAI’s potential for scalable, low-cost deployment in time-sensitive or resource-limited clinical settings.

Detailed latency distributions and cost-calculation parameters are provided in Supplementary Material SM8.

### Summary of findings

Overall, LingualAI preserved semantic accuracy at levels comparable to certified interpreters but showed limitations in delivery quality and expressiveness. Among the primary factors, both terminology accuracy and adequacy of meaning met the non-inferiority thresholds, confirming that the AI system conveyed medical content reliably.

Within the secondary factors, only completeness met the non-inferiority criterion, while grammar, vocabulary, and cultural appropriateness favored human interpreters. Notably, differences in vocabulary and cultural appropriateness reached significance primarily in patient statements (Spanish → English).

All voice-quality factors—fluency, prosody, and pacing showed large and consistent human advantages, underscoring the gap in naturalness and conversational rhythm.

Finally, both conclusive factors—overall quality and clinical confidence were significantly higher for human translations. Together, these findings suggest that while LingualAI effectively preserves meaning and accuracy, its synthesized speech still lacks the nuance and expressivity essential for fully natural clinical communication.

## Discussion

In this prospective, within-subject comparison of an AI-based translation tool (LingualAI) with certified medical interpreters, LingualAI preserved two primary dimensions—terminology accuracy and adequacy of meaning, at levels meeting prespecified non-inferiority thresholds. These results indicate reliable conveyance of core medical content. LingualAI also achieved non-inferiority for completeness, supporting its ability to maintain the overall structure and continuity of clinical dialogue.

In contrast, certified medical interpreters consistently outperformed LingualAI in several secondary and voice-related domains, including grammar, vocabulary, cultural appropriateness, fluency, prosody, and pacing. These findings highlight the continued importance of delivery quality in clinical communication. While semantic integrity was preserved, aspects critical to trust, rapport, and nuanced interaction remained stronger with professional interpreters. Notably, the dimensions most directly linked to clinical safety – meaning and terminology accuracy, were maintained, even as spoken delivery remained less natural than human interpretation.

These findings suggest that AI-based translation tools may have a role in supporting basic clinical comprehension, particularly in settings where interpreter services are unavailable or delayed. Such contexts may include urgent encounters, after-hours care, or resource-limited environments where proceeding without translation support could compromise care. From a practical perspective, accurate conveyance of meaning and terminology is often more critical than perfectly natural speech delivery in time-sensitive situations.

While human interpreters provide richer tone, empathy, and contextual nuance, LingualAI’s consistent semantic accuracy and scalability suggest potential value as an adjunct when access or affordability is constrained. Importantly, these findings do not support replacing certified medical interpreters. Rather, they indicate that current AI-based translation may extend access to essential communication when traditional services are temporarily unavailable.

The acceptability of AI-supported translation depends on alignment with established professional standards for medical interpretation. Collaboration with certified medical translation bodies is therefore essential. AI translation should be viewed as an assistive technology that expands access while preserving the central role of professional interpreters in ensuring safe, culturally appropriate, and patient-centered communication.

**Fig. 3:**
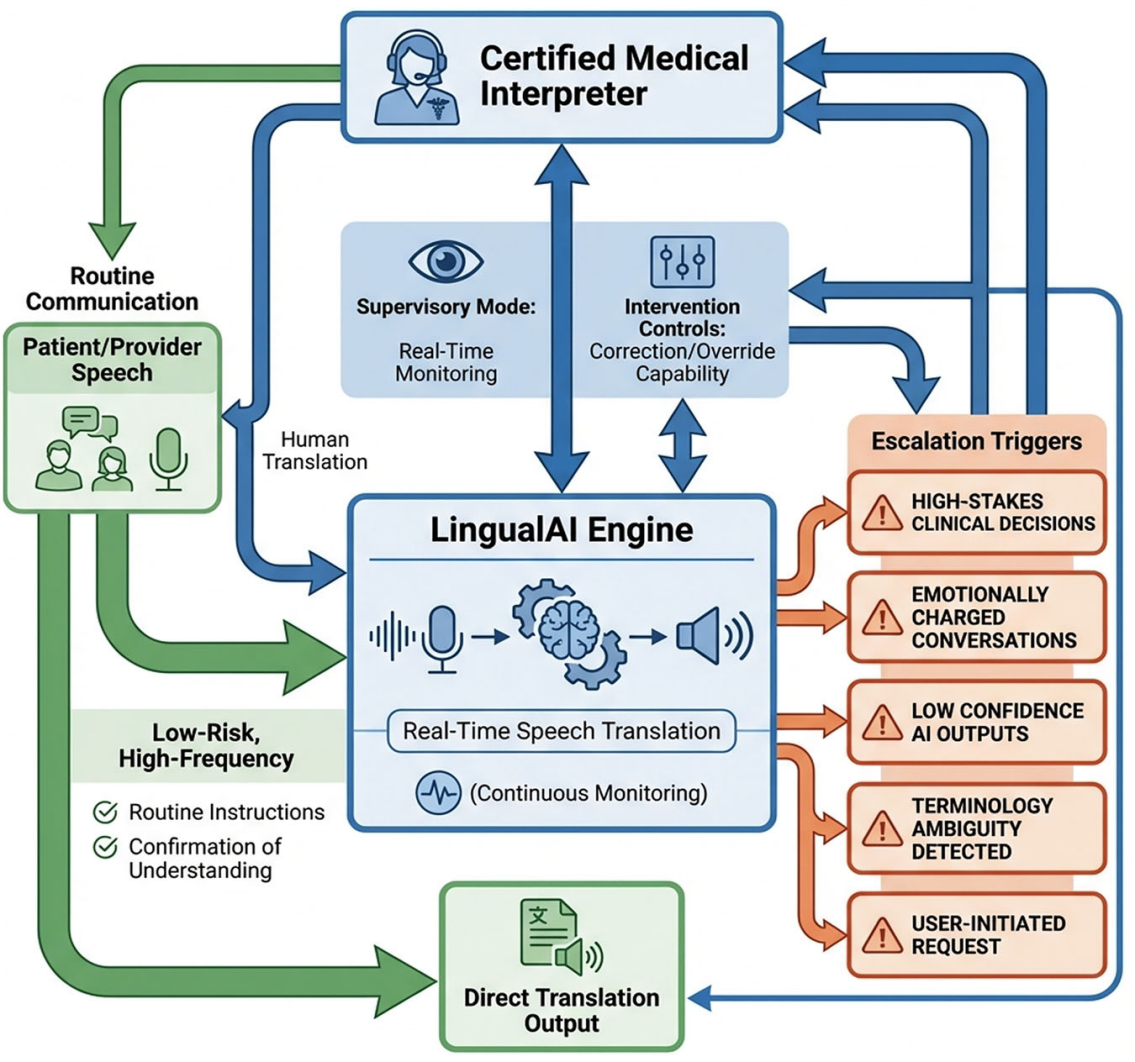
Proposed interpreter-in-the-loop model for AI-supported clinical translation. LingualAI is envisioned to provide continuous, real-time speech translation for low-risk, high-frequency clinical communication, such as routine instructions and confirmation of understanding. Certified medical interpreters are positioned in a supervisory role, with real-time visibility into AI-mediated exchanges and the ability to intervene through correction or override when appropriate. Predefined escalation triggers—including high-stakes clinical decisions, emotionally charged conversations, low-confidence AI outputs, detected terminology ambiguity, or user-initiated requests—are intended to prompt timely involvement of a certified interpreter. This proposed model aims to preserve semantic accuracy and patient safety while improving access, timeliness, and scalability of multilingual communication in clinical settings.

Based on the scope and findings of this study, we propose an interpreter-in-the-loop model for responsible clinical deployment of AI-based translation (Fig. 3). In this framework, LingualAI provides continuous, real-time translation for low-risk, high-frequency communication, such as routine instructions, clarification, or confirmation of understanding. Escalation to a certified medical interpreter is enabled for high-stakes clinical decisions, emotionally sensitive conversations, low-confidence AI outputs, detected terminology ambiguity, or user-initiated requests. Interpreters may operate in a supervisory role, monitoring AI-generated translations and intervening through correction or override when needed. This approach preserves professional accountability while improving the timeliness and scalability of multilingual communication.

Under Section 1557 of the Affordable Care Act and current guidance from the Office for Civil Rights, AI-based translation tools may support language access in healthcare, but generally cannot serve as the sole mechanism for ensuring meaningful access for individuals with LEP.^10,11^. Substantive clinical communications such as informed consent, treatment decisions, and discharge planning typically require involvement of certified human interpreters. AI systems may improve efficiency and terminology consistency, but their use should remain embedded within workflows that include appropriate human oversight.

Our findings are consistent with prior evaluations of general-purpose and medical translation tools, which commonly report strengths in terminology and adequacy of meaning alongside persistent weaknesses in contextual interpretation, cultural appropriateness, and delivery-related qualities. Studies of systems such as Google Translate have shown similar patterns, with reliable basic content translation but limitations in nuance, dialect sensitivity, and cultural context.^12,13^.

Work on large language models has highlighted limitations of largely literal translation approaches that may fail to capture nuances essential for medical communication^14,15^. Additional evidence suggests that AI-based translations can lack cultural awareness needed to support trust and rapport in diverse patient populations, potentially leading to misunderstandings^16^. Although some systems can generate fluent output, syntactic and semantic inconsistencies remain common, particularly in languages with complex grammatical structures.^17^.

This study extends existing literature by demonstrating that even a purpose-built, domain-specific system optimized for clinical use exhibits comparable strengths and limitations. Unlike many prior evaluations, our study employed a prospective, within-subject design with blinded bilingual clinician raters, multidomain scoring, and a prespecified non-inferiority framework. This approach strengthens confidence in the findings and clarifies which aspects of translation performance may be safely supported by AI and which continue to require human expertise.

This study has several strengths. The within-subject paired design enabled direct comparison between AI and human translations. Blinded evaluation by bilingual clinicians reduced overt source bias. The use of a structured, multidomain rubric and a prespecified non-inferiority framework allowed rigorous assessment across clinically relevant dimensions. Combining paired statistical tests with mixed-effects modeling further improved robustness of inference.

Several limitations warrant consideration. The evaluation used scripted scenarios within a single clinical specialty, which may not fully capture the variability of spontaneous clinical dialogue, including emotional expression, interruptions, and background noise encountered in real-world settings. The study focused on audio-based translation and did not evaluate text-based interfaces, which may influence comprehension differently. In addition, the study was conducted outside live clinical encounters and may not reflect the operational pressures of real-time care delivery.

Evaluator-related factors introduce additional limitations. Raters were anonymized, and participation varied across scenarios, resulting in an unbalanced dataset. Inter-rater reliability was modest (Krippendorff’s α = 0.31), reflecting a combination of factors rather than first-impression scoring. These include the absence of formal rater calibration, domain-specific subjectivity, particularly for perceptual and voice-related constructs such as naturalness, fluency, and clinical confidence, and heterogeneous perceptual thresholds across clinicians. Such variability likely reduces agreement even when evaluators broadly concur on relative performance differences between AI and human translations. Consequently, comparisons near the non-inferiority margin, especially for subjective delivery-related domains, should be interpreted with appropriate caution. Future evaluations should incorporate structured rater calibration and brief training using exemplar cases, particularly for perceptual domains, as well as larger and more balanced evaluator panels to improve inter-rater reliability.

Although evaluations were nominally blinded, raters may have inferred translation source based on synthetic voice characteristics or delivery style. Such partial unblinding would be expected to disadvantage AI performance rather than inflate it. Accordingly, the observed non-inferiority findings for meaning and terminology accuracy should be interpreted as conservative estimates of semantic performance.

Additionally, this study evaluated English–Spanish translation only, and results may not generalize to other language pairs or dialects. Performance may vary substantially across linguistic contexts, particularly those with different grammatical structures or sociolinguistic norms.

Future development of LingualAI should prioritize improvements in speech synthesis, particularly prosody, pacing, and fluency, which showed the largest performance gaps relative to certified interpreters. Validation in live, unscripted clinical encounters will be essential to assess performance under real-world conditions. Incorporating patient perspectives and expanding evaluation to additional languages and dialects will further strengthen generalizability.

LingualAI demonstrated non-inferior performance in preserving meaning and terminology, the core dimensions of translation accuracy. Certified medical interpreters remained superior in delivery-related qualities, including fluency, prosody, and clinical confidence. These findings support a role for AI-based translation as a supplementary tool when interpreter access is constrained, provided use remains aligned with professional standards. An interpreter-in-the-loop approach offers a responsible pathway for deployment, with human expertise ensuring safety, nuance, and patient trust as translation technologies continue to evolve.

## Methods

We conducted a prospective, within-subject simulation-based comparison of translations generated by the LingualAI application versus certified medical interpreters (Fig. 2). Two translation directions were evaluated: English → Spanish (clinician utterances) and Spanish → English (patient utterances). Each input phrase was translated by both arms, enabling paired comparisons with evaluators blinded to translation source but not scenario context. Similar prospective, paired or randomized designs have been used in prior translation and interpreter studies that have compared Machine or AI Translation with certified medical interpreters and bilingual clinical evaluators^18,19^.

### Clinical scenarios and audio collection

Three standardized otorhinolaryngology scenarios were developed to represent typical care interactions. Together, the scripts included 18 dialogue paragraphs (33 lines) covering history-taking, clinical instructions, and empathic statements. Each scenario was enacted by two pairs of native English and Spanish speakers to reduce bias from voice quality or pronunciation differences. As a result, each phrase was recorded twice, yielding two independent input sets. Use of standardized scripted encounters with native speakers mirrors prior interpreter research and simulation-based translation evaluations^20,21^. The full text of the three standardized scenarios, together with descriptive information on dialogue length, speaker role, and clinical terminology content, is provided in SM1.

### Translation pipelines

In the AI arm, recordings were processed through LingualAI, which integrates automatic speech recognition (ASR), neural machine translation (NMT), and text-to-speech synthesis. Each phrase was submitted twice using the two independent source recordings, generating two AI-translated audio files per phrase in each direction. Outputs were pooled and randomized in blocks of 2 using a fixed random seed to ensure reproducibility, and evaluators were blinded to the translation source (AI vs. human). They were, however, provided with scenario context, line order, and speaker role (clinician vs. patient) so that ratings reflected the intended clinical meaning. Modular pipelines combining ASR, NMT, and synthesis are well described in biomedical translation research^7,22,23^. This evaluation was intentionally designed to assess end-to-end clinical speech-to-speech translation under deployment-realistic privacy constraints. Because LingualAI operates on protected health information and real-time audio streams that cannot be exported to public corpora, we used certified medical interpreters as the clinical reference standard rather than public text-translation benchmarks. Automatic text-based metrics such as BLEU do not capture clinically relevant failure modes in speech-to-speech translation, including automatic speech recognition errors, timing and latency effects, turn-taking disruption, and synthesized speech naturalness. For this reason, certified medical interpreters were selected as the clinical reference standard for evaluating end-to-end clinical translation quality. This choice reflects real-world clinical deployment constraints, where safety, meaning preservation, and delivery quality are judged relative to professional interpretation rather than automated metrics.

LingualAI’s pipeline operates in five real-time stages: (i) voice capture, (ii) text conversion via ASR, (iii) refinement using prior dialogue and domain-specific vocabulary, (iv) translation into the target language, and (v) speech synthesis for natural-sounding delivery. Each stage is designed for real-time performance with fallback strategies in case of network interruptions. The most recent update includes support for custom vocabularies, allowing clinicians to register specialized or uncommon medical terms to improve transcription and translation accuracy in clinical contexts. Comparable staged architectures with domain adaptation have been reported for ambient clinical transcription and multilingual MT systems^22^.

In the human arm, two certified medical interpreters independently translated the scenarios, producing two human-translated audio files per phrase. Outputs were similarly pooled and randomized to reduce interpreter-specific bias. All translations were recorded under uniform audio conditions.

### Evaluators

Nine fluent bilingual clinicians (English–Spanish) served as evaluators. Participation in the evaluation was voluntary and completed outside protected clinical time. Evaluators submitted ratings anonymously; unique rater identifiers were not collected. Audio clips were randomized and presented blinded to translation source (AI vs. human), but with scenario context provided to support intended meaning. Although blinding was applied, evaluators may have inferred AI-generated outputs due to the use of a consistent synthetic voice and comparatively mechanistic delivery, particularly in voice-related domains. We did not conduct a calibration round, as the study was designed to capture independent first-impression ratings rather than consensus scoring, a choice that likely contributed to modest inter-rater agreement in perceptual and delivery-related domains. All evaluators provided Consent to Participate prior to beginning the study procedures. Blinded bilingual rater designs have been applied in comparable translation and medical communication studies^18,24^.

### Rating instrument

Translation quality was assessed across 12 quality domains: (i) primary: adequacy of meaning, terminology accuracy; (ii) secondary: completeness, cultural appropriateness, grammar, vocabulary; (iii) voice-related: fluency, clarity, prosody, pacing; and (iv) conclusive: overall quality and clinical confidence. Ratings used 5-point Likert scales (1 = poor, 5 = excellent), and evaluators were provided with a guide containing definitions and examples. Evaluators rated each clip independently using Google Forms, a secure, web-based platform containing domain definitions and illustrative examples. Similar multidomain rubrics have been applied in medical machine translation and LLM evaluation studies^18,19^. Established frameworks such as the Multidimensional Quality Metrics (MQM) cover accuracy, fluency, terminology, and adequacy, with error taxonomies also informing clarity, grammar, and overall quality^25^. In line with broader recommendations, our rubric emphasized context awareness, distinguishing accuracy from fluency, and incorporating completeness, cultural appropriateness, and clinical relevance into evaluation.^26,27^. Additionally, because of this we did not conduct a calibration round, as the study was designed to capture first-impression ratings rather than consensus. The complete rating instrument and evaluator instructions are provided in Supplementary Material SM2.

### Statistical analysis

Normality of scores was assessed using the Shapiro–Wilk test. For normally distributed outcomes, paired t-tests were applied; for non-normal distributions, Wilcoxon signed-rank tests were used. Effect sizes were reported as Cohen’s d (t-test) or correlation coefficient (r) (Wilcoxon). Inter-rater reliability was estimated using Krippendorff’s α, which accommodates missing data and unequal numbers of raters per item, a necessity given that evaluators submitted ratings anonymously without unique identifiers. Mixed-effects linear models with random intercepts for item (line) were applied to account for repeated ratings of the same line across multiple evaluators.

A non-inferiority margin of 0.30 points on the 5-point scale was prespecified. This margin was chosen a priori based on three considerations: (i) the half-SD rule commonly applied to Likert ratings, where typical standard deviations of ~0.5–0.7 correspond to a minimal meaningful difference of ~0.25–0.35; (ii) prior studies that used similar small margins for clinically relevant differences on 5-point rating scales^28^; and (iii) expert consensus among bilingual clinician-evaluators that a 0.30 shift would begin to alter clinical confidence without changing literal meaning. For each domain, one-sided 95% confidence intervals (CIs) were calculated for the mean difference between human and AI ratings. LingualAI was considered non-inferior if the upper bound of the CI was less than the margin. All analyses were conducted in Python (Ver 3.12.5 using Pandas, NumPy, statsmodels and SciPy libraries) and R (version 4.4.2 using ggplot2, dplyr, tibble libraries), with statistical significance set at two-sided *p* < 0.05. This non-inferiority framework is consistent with prior comparative translation studies^9,28,29^.

For a priori power, we planned for 33 lines, each rated by ~7 bilingual clinicians (≈231 ratings). Because ratings on the same line are correlated, we adjusted for clustering using an assumed intraclass correlation (ICC) of 0.25, giving an effective sample size of ${n}_{\mathrm{eff}}=\frac{33\times 7}{1+(7-1)\times 0.25}\approx 92$. With a one-sided non-inferiority test (α = 0.025), a prespecified margin of Δ = 0.30, and an expected true mean difference of 0.10, the projected power was ~89% (Supplementary Material SM3). Sensitivity checks varying ICC (0.20–0.30) and score variability (SD 0.60–0.70) yielded power between ~74% and 93%, supporting the adequacy of the planned sample size.

### Exploratory system metrics

In addition to translation quality, we recorded system-level performance metrics, including end-to-end translation latency and estimated operational cost. Latency was computed from system logs as the total time between audio input and synthesized output, encompassing automatic speech recognition, neural translation, and text-to-speech synthesis. Operating cost was estimated based on API usage and computational overhead for a 10-min bidirectional conversation, using unit prices of deployed components. These exploratory analyses aimed to contextualize LingualAI’s technical efficiency and cost-effectiveness relative to conventional interpreter services.

### Human ethics and consent to participate

This study was reviewed by the UTHealth Houston Committee for the Protection of Human Subjects (CPHS) and was determined not to meet the regulatory definition of human subjects research; therefore, further Institutional Review Board (IRB) review was not required (Reference No. HSC-SBMI-26-0139; IRIS Ref. 287958).

All clinician evaluators participated on a voluntary basis and provided informed consent prior to engaging in any study-related activities. The study was conducted in accordance with the ethical principles outlined in the Declaration of Helsinki.

No patients or members of the public were recruited or enrolled as participants. Certified medical interpreters provided translation services in their professional capacity and were compensated for their work; they were not considered study participants.

## Supplementary information

Supplementary Material 1-8, LingualAI.

## Data Availability

De-identified audio clips and raw evaluator rating data underlying this study are available from the corresponding author upon reasonable request. Access will require completion of a data-use agreement and approval by the appropriate Institutional Review Board. Summary-level data (means, standard deviations, and confidence intervals) are included in the main manuscript and Supplementary Materials. Due to file size, PHI redaction requirements, and copyright considerations, raw audio files and individual rating sheets are not publicly posted.
