# Supplementary material for: Evaluating LingualAI: a prospective validation of AI-based real-time translation against certified human interpreters
Source: Npj Health Syst. 2026 May 12;3:29. doi: 10.1038/s44401-026-00080-5 (PMC13354185; doi:10.1038/s44401-026-00080-5)
Supplement: Supplementary file 1 — Supplementary Material 1-8, LingualAI. [file 44401_2026_80_MOESM1_ESM.pdf]

SUPPLEMENTARY MATERIAL 1 (SM1)

Title	Short 1-sentence summary	Clinical setting & intent	No of dialogues	Clinical terminology density*	Linguistic characteristics	Accent / dialect information
Post-Surgical Check-Up (Sinus Surgery)	Routine postoperative outpatient follow-up addressing persistent nasal symptoms and treatment adherence after sinus surgery.	Otolaryngology outpatient follow-up; symptom assessment, medication adherence, reassurance, and follow-up planning	~13 lines, mean ~17 words per line	Moderate (e.g., *Budesonide, inflammation, eustachian tubes, nasal steroid spray*)	Mixed short and moderate-length utterances; descriptive symptom language; instructional counseling	Native English-speaking clinician; native Spanish-speaking patient (standard Latin American Spanish)
Medication and Side Effects	Medication reconciliation visit focusing on chronic disease management and adverse effects from antihypertensive and diabetic medications.	Primary care / outpatient management; medication adherence, side-effect evaluation, and treatment adjustment	~11 lines, mean ~14 words per line	Moderate-to-high (e.g., *Losartan, Metformin, blood sugar, diabetes*)	Medication-focused vocabulary; explanatory counseling; cause-effect reasoning	Native English-speaking clinician; native Spanish-speaking patient (standard Latin American Spanish)
Emergency Room Visit – Chest Pain	Acute emergency encounter evaluating chest pain with concern for possible cardiac event.	Emergency department; rapid triage, risk assessment, and urgent diagnostic decision-making	~11 lines, mean ~10 words per line	High (e.g., *EKG, blood tests, heart attack, high blood pressure*)	Short, urgent utterances; closed-ended questioning; time-sensitive language	Native English-speaking clinician; native Spanish-speaking patient (standard Latin American Spanish)

Scenarios 1-3 used for validation of LingualAI

Scenario 1: Post-Surgical Check-Up (Sinus Surgery)

Doctor: Hi, it's good to see you again. How have you been doing since the surgery?
Patient: Creo que estoy bien, pero mi nariz aún se siente hinchada y tengo algo de congestión. A veces me cuesta respirar por la nariz.
Doctor: That's expected. The swelling should go down over the next few weeks. Are you still doing the Budesonide irrigations?
Patient: No, dejé de hacerlo la semana pasada.
Doctor: That might be why your symptoms are coming back. The irrigations help keep inflammation down. I'd like you to restart them twice a day.
Patient: Está bien. Además, siento algo de presión en la frente y a veces tambien siento que mis oídos están tapados.
Doctor: That could be from lingering inflammation in your sinuses. Let me take a look. Everything looks like it's healing well. No signs of infection. But I do see some fluid behind your eardrums, which can happen when the sinuses are inflamed.
Patient: Entonces, ¿ese problema se resolverá solo?
Doctor: Usually, yes. If it doesn't, we might need to try a nasal steroid spray to help open up the eustachian tubes. Let's see how you feel over the next month.
Patient: Está bien.
Doctor: We'll check on you again in six weeks. If anything worsens before then—like severe pain, bleeding, or fever, call us right away.

Scenario 2: Medication and Side Effects

Doctor: Are you still taking all your prescribed medications?
Patient: Creo que sí. Tomo uno por la mañana y otro por la noche.
Doctor: Let's go over them. You're on Losartan for blood pressure, Metformin for diabetes, and Budesonide for your nose. Are you taking all of them as directed?
Patient: Tomo Losartan cada mañana, pero dejé de tomar Metformina porque me hacía sentir mareado.
Doctor: When did you stop?
Patient: Hace dos semanas.
Doctor: That could be why your blood sugar is higher. Metformin doesn't usually cause dizziness. Have you been checking your blood sugar levels?
Patient: No, no tengo un monitor.
Doctor: I'll arrange for you to get one. Also, stopping Metformin suddenly can cause issues. Let's start it again, but we'll lower the dose to see if that helps.
Patient: Está bien. Además, me siento con mucha sed todo el tiempo.
Doctor: That's another sign your blood sugar might be too high. We'll run a blood test today to check your levels.

Scenario 3: Emergency Room Visit – Chest Pain

Doctor: What brought you to the ER today?
Patient: Esta mañana empecé a sentir dolor en el pecho. Se siente como una fuerte presión.
Doctor: Does the pain spread to your arm, neck, or jaw?
Patient: Mi brazo izquierdo se siente un poco entumecido.

Doctor: Are you short of breath? Feeling nauseous or sweaty?
Patient: Sí, empecé a sudar mucho y me siento un poco mareado.
Doctor: This could be a heart issue. We're going to do an EKG and blood tests right away. Have you had chest pain like this before?
Patient: No, nunca.
Doctor: Do you have a history of high blood pressure, diabetes, or heart disease?
Patient: Tengo presión alta, pero no siempre me tomo mi medicación.
Doctor: That might be contributing. Let's get these tests done quickly. If it is a heart attack, we need to act fast.

SUPPLEMENTARY MATERIAL 2 (SM2)

Rating Instrument

Home Page of the Google Form for each scenario:
Welcome to the LingualAI Evaluation Study.

Thank you for participating in this study.

This questionnaire is designed to capture your preferences regarding translations that enable a bilingual (English and Spanish) conversation between a patient and a clinician. The conversation consists of a few paragraphs (reflecting a two-way exchange: questions/statements and responses).

You will first hear the original conversation between the doctor and the patient, followed by two separate translations—one generated by AI and one by a certified, experienced translator. To maintain impartiality, we have not labelled which translation is which. We kindly ask that you set aside any pre-existing opinions you may have about the general capabilities of AI or human translators.

For each translation, you will evaluate it based on 12 parameters, which are detailed on each page. Please note that all your responses are completely anonymous, and no identifying information will be linked to your feedback.

Project Team
Leadership
Project ideation: Dr. Xiaoqian Jiang
Project facilitation: Dr. Babatope O Fatuyi
End-to-End App development: Carlos A Jaimes Garcia
UI/UX expert: Cecilia Wang
Evaluation Coordinator: Uday Pratap Singh

Contacts: Jingqi.Wang.1@uth.tmc.edu, uday.pratapsingh@uth.tmc.edu

Thank you again for your participation and valuable feedback.

—-

Line 01 (Clinician) - Original Statement
🎧 Listen the original audio spoken by the Clinician during the consultation: Drive Link

Line 01 (Clinician) - Translation A
Listen to Translation A: Drive Link

Please evaluate this translation based on the original speaker’s intent and clarity.

Score 1 (very poor) through 5 (Excellent) for the following:

- Adequacy (Accuracy of Meaning): Does the audio rendition fully and faithfully convey the meaning of the source message, without omissions or additions?
- Terminology Accuracy: Are specialized clinical and medical terms (e.g., drug names, procedures, diagnoses) correctly and precisely translated?
- Fluency / Naturalness: Does the phrasing sound smooth, natural, and native-like in the target language, avoiding awkward or literal constructions?
- Intelligibility (Clarity of Speech): Is every word easy to hear and understand due to proper pronunciation and clear audio quality?
- Prosody & Tone: Is the intonation, rhythm, and tone of voice appropriate for a professional clinical setting?
- Pacing (Speed & Rhythm): Is the speaking rate comfortable to follow: not too fast, not too slow, for a clinical listener?
- Completeness: Does the audio include all critical information from the original, without introducing unnecessary content?
- Cultural & Contextual Appropriateness: Are cultural references, idioms, and phrasing adapted appropriately for the patient’s background and clinical context?
- Grammar & Syntax: Is the audio grammatically correct, with proper sentence structure and word order in the target language?
- Vocabulary (General Language Use): Are common, appropriate, and consistent words chosen across the message (not overly technical or simplistic)?
- Overall Quality: Overall, how would you rate this audio as a clinical communication tool?
- Confidence for Clinical Use: If this translation were used in a real clinical interaction, how confident would you feel relying on it?

Line 01 (Clinician) - Translation B
Listen to Translation B: Drive Link

Please evaluate this translation based on the original speaker’s intent and clarity.

Score 1 (very poor) through 5 (Excellent) for the following:

- Adequacy (Accuracy of Meaning): Does the audio rendition fully and faithfully convey the meaning of the source message, without omissions or additions?
- Terminology Accuracy: Are specialized clinical and medical terms (e.g., drug names, procedures, diagnoses) correctly and precisely translated?
- Fluency / Naturalness: Does the phrasing sound smooth, natural, and native-like in the target language, avoiding awkward or literal constructions?
- Intelligibility (Clarity of Speech): Is every word easy to hear and understand due to proper pronunciation and clear audio quality?

- Prosody & Tone: Is the intonation, rhythm, and tone of voice appropriate for a professional clinical setting?
- Pacing (Speed & Rhythm): Is the speaking rate comfortable to follow: not too fast, not too slow, for a clinical listener?
- Completeness: Does the audio include all critical information from the original, without introducing unnecessary content?
- Cultural & Contextual Appropriateness: Are cultural references, idioms, and phrasing adapted appropriately for the patient's background and clinical context?
- Grammar & Syntax: Is the audio grammatically correct, with proper sentence structure and word order in the target language?
- Vocabulary (General Language Use): Are common, appropriate, and consistent words chosen across the message (not overly technical or simplistic)?
- Overall Quality: Overall, how would you rate this audio as a clinical communication tool?
- Confidence for Clinical Use: If this translation were used in a real clinical interaction, how confident would you feel relying on it?

Similarly for each line, 1. Original Statement, then Translation A (Certified human translator or LingualAI) followed by Translation B (Certified human translator or LingualAI)

SUPPLEMENTARY MATERIAL 3 (SM3)

A-priori power calculation

```
=== A Priori Non-Inferiority Power (with clustering) ===

“Given our design and expected variability, do we have enough statistical power
(≥80%) to detect that LingualAI is not worse than human translators by more than Δ =
0.30 points?”

Inputs: N_LINES=33, MEAN_RATERS=7.0, ICC=0.25
        DELTA=0.30, MU_TRUE=0.10, SD_DIFF=0.60, ALPHA=0.025

Effective sample size (n_eff): 92.40
One-sided NI power: 89.3%

Sensitivity (power %) across ICC and SD assumptions:
 ICC  |  SD   |  n_eff  |  Power%
------------------------------------
 0.20 | 0.60 |  105.0 |   92.7
 0.20 | 0.65 |  105.0 |   88.4
 0.20 | 0.70 |  105.0 |   83.3
 0.25 | 0.60 |   92.4 |   89.3
 0.25 | 0.65 |   92.4 |   84.1
 0.25 | 0.70 |   92.4 |   78.4
 0.30 | 0.60 |   82.5 |   85.7
 0.30 | 0.65 |   82.5 |   79.8
 0.30 | 0.70 |   82.5 |   73.7

Using a one-sided non-inferiority test (α=0.025), Δ=0.30, σ_d=0.60, expected
μ_d=0.10, and clustering adjustment with mean 7 raters/line and ICC=0.25 (n_eff≈92),
the projected power was 89%.
```

Python Code:

```
# Non-inferiority a priori power calculator with clustering (no external deps)
# -------------------------------------------------------------------------
# This script computes:
#  1) Effective sample size after clustering by line/items (design effect)
#  2) One-sided non-inferiority power using a normal approximation
#  3) A small sensitivity table across plausible ICC and SD values
#  4) A ready-to-paste Methods sentence
#
# Assumptions (edit the PARAMETERS block below to fit your study):
#  - Non-inferiority margin Δ on a 5-point Likert scale
```

```
#  - Expected true mean difference μ_d (human - AI), positive favors humans
#  - SD of paired differences σ_d (often ~0.6–0.7 for Likert domain deltas)
#  - Raters are clustered within items (lines), accounted via ICC and design effect
#
# No SciPy required. Uses a z-alpha lookup for alpha ∈ {0.05, 0.025, 0.01}.

from math import sqrt, erf

# -------------------- PARAMETERS --------------------
N_LINES = 33                # Total items/lines
MEAN_RATERS = 7.0           # Average raters per line
ICC = 0.25                  # Intraclass correlation among raters on the same line
DELTA = 0.30                # Non-inferiority margin Δ (points on a 5-point scale)
MU_TRUE = 0.10              # Expected true mean difference (human - AI)
SD_DIFF = 0.60              # SD of paired differences σ_d
ALPHA = 0.025               # One-sided alpha (use 0.025 for 95% NI CI)
ICC_GRID = [0.20, 0.25, 0.30]
SD_GRID  = [0.60, 0.65, 0.70]
# ------------------------------------------------------------

# Normal CDF via error function
def Phi(z: float) -> float:
    return 0.5 * (1.0 + erf(z / sqrt(2.0)))

# Common z-quantiles for one-sided alpha
Z_ALPHA_LOOKUP = {
    0.05:  -1.6448536269514722,
    0.025: -1.959963984540054,
    0.01:  -2.3263478740408408,
}

def z_alpha(alpha: float) -> float:
    if alpha in Z_ALPHA_LOOKUP:
        return Z_ALPHA_LOOKUP[alpha]
    # Fallback: simple linear interpolation between nearby keys (coarse but fine for planning)
    keys = sorted(Z_ALPHA_LOOKUP.keys())
    if alpha <= keys[0]:
        return Z_ALPHA_LOOKUP[keys[0]]
    if alpha >= keys[-1]:
        return Z_ALPHA_LOOKUP[keys[-1]]
    # interpolate
    for i in range(len(keys)-1):
        if keys[i] <= alpha <= keys[i+1]:
            a0, a1 = keys[i], keys[i+1]
```

```
        z0, z1 = Z_ALPHA_LOOKUP[a0], Z_ALPHA_LOOKUP[a1]
        t = (alpha - a0) / (a1 - a0)
        return z0 + t * (z1 - z0)

def effective_n(n_items: int, mean_raters_per_item: float, icc: float) -> float:
    """
    Design effect for cluster (item)-level clustering with m raters per item and ICC=icc.
    n_eff = (n_items * m) / (1 + (m - 1) * ICC)
    """
    m = mean_raters_per_item
    deff = 1.0 + (m - 1.0) * icc
    return (n_items * m) / deff

def power_noninferiority(delta: float,  # NI margin Δ
                         mu_true: float,  # expected true mean difference μ_d (human - AI)
                         sd_diff: float,  # SD of paired differences
                         n_eff: float,    # effective sample size after clustering
                         alpha: float = 0.025):
    """
    One-sided NI power using normal approximation.
    Reject H0 if T < z_alpha, where T ~ N( (μ_d - Δ)/(σ/√n_eff), 1 ).
    Power = Φ( z_alpha - (μ_d - Δ)/(σ/√n_eff) )
    """
    za = z_alpha(alpha)
    ncp = (mu_true - delta) / (sd_diff / sqrt(n_eff))
    return Phi(za - ncp)

def methods_sentence(n_lines, mean_raters, icc, delta, mu_true, sd_diff, alpha):
    n_eff = effective_n(n_lines, mean_raters, icc)
    pw = power_noninferiority(delta, mu_true, sd_diff, n_eff, alpha)
    return (
        f"Using a one-sided non-inferiority test (α={alpha:.3f}), Δ={delta:.2f}, "
        f"σ_d={sd_diff:.2f}, expected μ_d={mu_true:.2f}, and clustering adjustment "
        f"with mean {mean_raters:.0f} raters/line and ICC={icc:.2f} (n_eff≈{n_eff:.0f}), "
        f"the projected power was {pw*100:.0f}%."
    )

# ---- Compute baseline estimates ----
n_eff_base = effective_n(N_LINES, MEAN_RATERS, ICC)
power_base = power_noninferiority(DELTA, MU_TRUE, SD_DIFF, n_eff_base, ALPHA)
sentence = methods_sentence(N_LINES, MEAN_RATERS, ICC, DELTA, MU_TRUE, SD_DIFF,
ALPHA)

print("=== A Priori Non-Inferiority Power (with clustering) ===\n")
```

```
print(f"Inputs: N_LINES={N_LINES}, MEAN_RATERS={MEAN_RATERS}, ICC={ICC:.2f}")
print(f"        DELTA={DELTA:.2f}, MU_TRUE={MU_TRUE:.2f}, SD_DIFF={SD_DIFF:.2f},
ALPHA={ALPHA:.3f}\n")
print(f"Effective sample size (n_eff): {n_eff_base:.2f}")
print(f"One-sided NI power: {power_base*100:.1f}%\n")

# ---- Sensitivity grid ----
print("Sensitivity (power %) across ICC and SD assumptions:")
print(" ICC  |  SD   |  n_eff  |  Power% ")
print("------------------------------------")
for icc in ICC_GRID:
    n_eff_g = effective_n(N_LINES, MEAN_RATERS, icc)
    for sd in SD_GRID:
        p = power_noninferiority(DELTA, MU_TRUE, sd, n_eff_g, ALPHA)
        print(f" {icc:0.2f} | {sd:0.2f} | {n_eff_g:6.1f} | {p*100:6.1f}")

# ---- Suggested Methods sentence ----
print("\nSuggested Methods sentence:")
print(sentence)
```

SUPPLEMENTARY MATERIAL 4 (SM4)

Stratified Results for Clinician and Patient Statements

Clinician statements (English → Spanish)

Primary factors: No significant differences were detected for adequacy of meaning (human 4.77 vs. app 4.71; $p = 0.41$) or terminology accuracy (4.70 vs. 4.68; $p = 0.81$). In contrast, clarity was significantly higher for human translations (4.87 vs. 4.27; $p = 0.003$, $r = 0.68$).

Secondary factors: Small, inconsistent effects were seen. Completeness did not differ (4.85 vs. 4.75; $p = 0.19$), while cultural appropriateness showed a moderate human advantage (4.84 vs. 4.42; $p < 0.001$, $r = 0.50$). Neither grammar (4.84 vs. 4.64; $p = 0.11$) nor vocabulary (4.78 vs. 4.61; $p = 0.20$) reached significance.

Voice quality related factors: Large differences favored human interpreters for fluency (4.82 vs. 3.63; $p < 0.001$, $r = 0.77$), prosody (4.90 vs. 4.28; $p < 0.001$, $r = 0.76$), and pacing (4.87 vs. 4.31; $p < 0.001$, $r = 0.72$).

Conclusive factors: Both overall quality (4.75 vs. 4.17; $p < 0.001$, $r = 0.60$) and confidence for clinical use (4.77 vs. 4.15; $p < 0.001$, $r = 0.61$) were significantly higher for human translations.

Patient statements (Spanish → English)

Primary factors: No differences were observed in adequacy of meaning (4.87 vs. 4.68; $p = 0.07$) or terminology accuracy (4.95 vs. 4.83; $p = 0.16$). Clarity was significantly higher for human translations (4.88 vs. 4.47; $p = 0.011$, $r = 0.58$).

Secondary factors: Human interpreters scored higher on completeness (4.89 vs. 4.72; $p = 0.12$, not significant), cultural appropriateness (4.95 vs. 4.58; $p < 0.001$, $r = 0.44$), grammar (4.93 vs. 4.72; $p < 0.001$, $r = 0.47$), and vocabulary (4.94 vs. 4.75; $p < 0.001$, $r = 0.48$).

Voice quality related factors: Significant human advantages were observed for fluency (4.91 vs. 3.82; $p = 0.002$, $r = 0.72$), prosody (4.87 vs. 4.32; $p < 0.001$, $r = 0.61$), and pacing (4.88 vs. 4.64; $p = 0.050$, $r = 0.45$).

Conclusive factors: Both overall quality (4.89 vs. 4.32; $p = 0.002$, $r = 0.70$) and confidence for clinical use (4.88 vs. 4.29; $p = 0.006$, $r = 0.64$) were significantly higher for human interpreters.

SUPPLEMENTARY MATERIAL 5 (SM5)

Domain category	Quality domain	n (paired ratings)	Mean difference (Human − AI)	Lower bound of one-sided 95% CI	Upper bound of one-sided 95% CI	Non-inferiority margin (Δ)	Non-inferior	Non-inferiority p-value (one-sided)	Paired p-value (two-sided)
**Primary factors**	Terminology accuracy	30	0.05	−0.07	0.17	0.20	**Yes**	0.024	0.461
**Primary factors**	Adequacy of meaning	30	0.14	0.03	0.25	0.20	No	0.193	0.044

**Primary factors**	Clarity / intelligibility	30	0.58	0.32	0.84	0.20	No	0.991	<0.001
**Secondary factors**	Completeness	30	0.18	0.06	0.30	0.20	No	0.391	0.018
**Secondary factors**	Grammar	30	0.22	0.07	0.36	0.20	No	0.586	0.016
**Secondary factors**	Vocabulary	30	0.18	0.02	0.34	0.20	No	0.426	0.063
**Secondary factors**	Cultural appropriateness	30	0.46	0.16	0.77	0.20	No	0.924	0.015
**Voice-related factors**	Fluency	30	1.18	0.74	1.62	0.20	No	0.999	<0.001
**Voice-related factors**	Pacing	30	0.47	0.30	0.65	0.20	No	0.994	<0.001
**Voice-related factors**	Prosody	30	0.72	0.46	0.98	0.20	No	0.999	<0.001
**Conclusive factors**	Overall quality	30	0.66	0.39	0.94	0.20	No	0.996	<0.001
**Conclusive factors**	Clinical confidence	30	0.72	0.44	1.01	0.20	No	0.998	<0.001

SUPPLEMENTARY MATERIAL 6 (SM6)

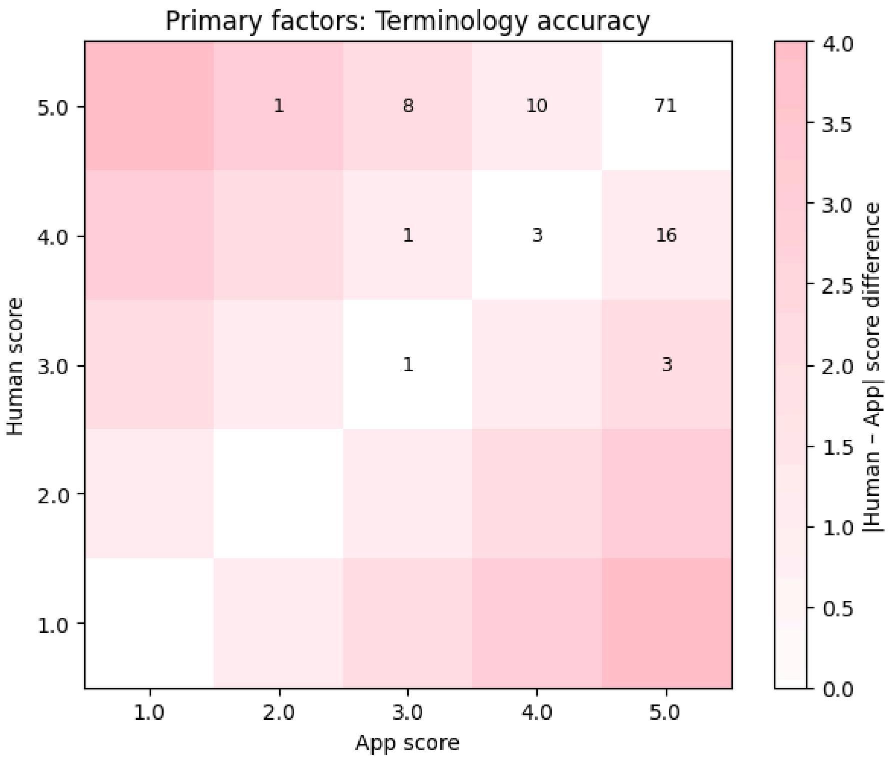

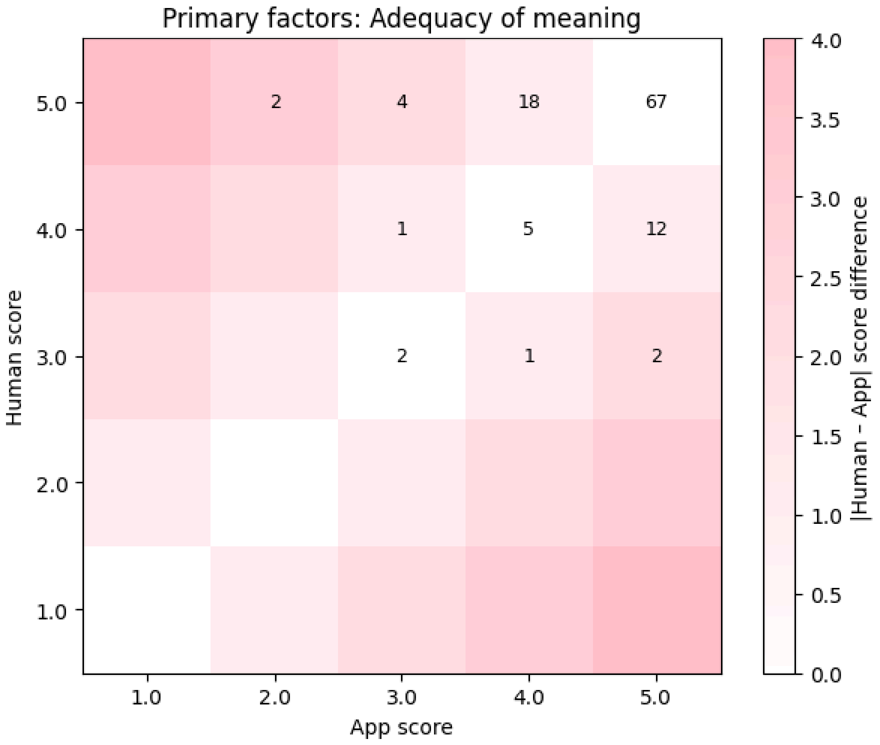

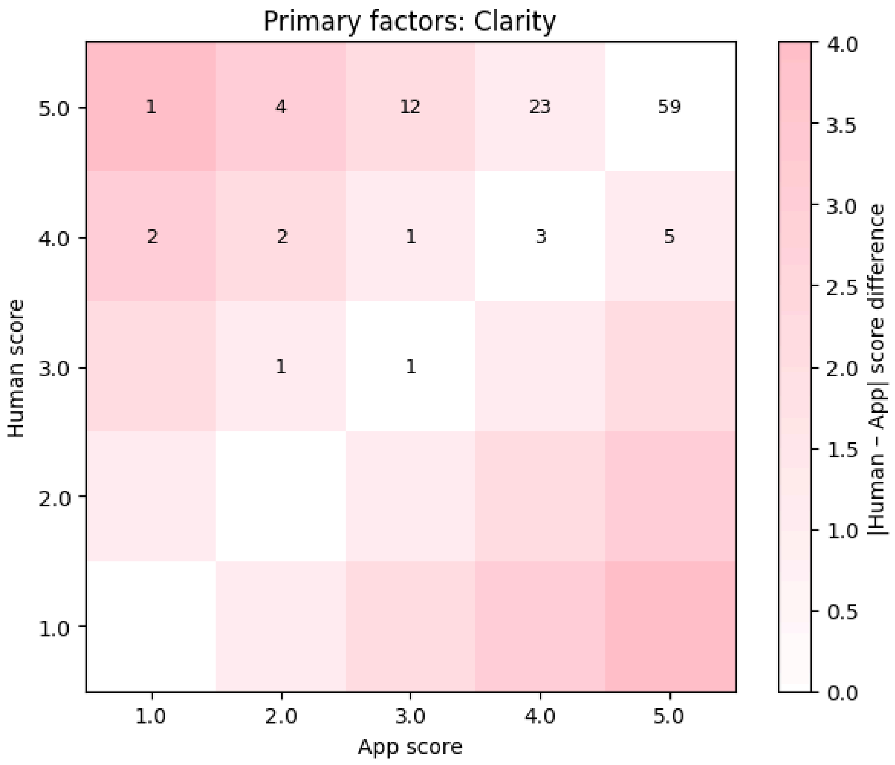
Primary factors: Clarity
1
4
12
23
59
2
2
1
3
5
1
1
5.0
4.0
3.0
2.0
1.0
Human score
1.0
2.0
3.0
4.0
5.0
App score
4.0
3.5
3.0
2.5
2.0
1.5
1.0
0.5
0.0
|Human – App| score difference

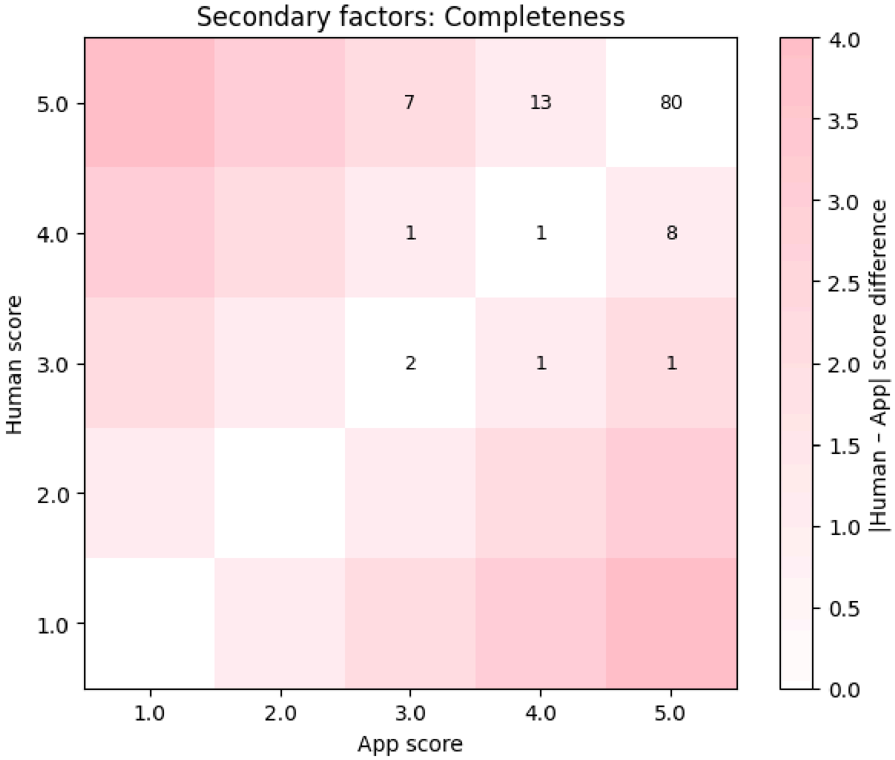
Secondary factors: Completeness
7
13
80
1
1
8
2
1
1
5.0
4.0
3.0
2.0
1.0
Human score
1.0
2.0
3.0
4.0
5.0
App score
4.0
3.5
3.0
2.5
2.0
1.5
1.0
0.5
0.0
|Human – App| score difference

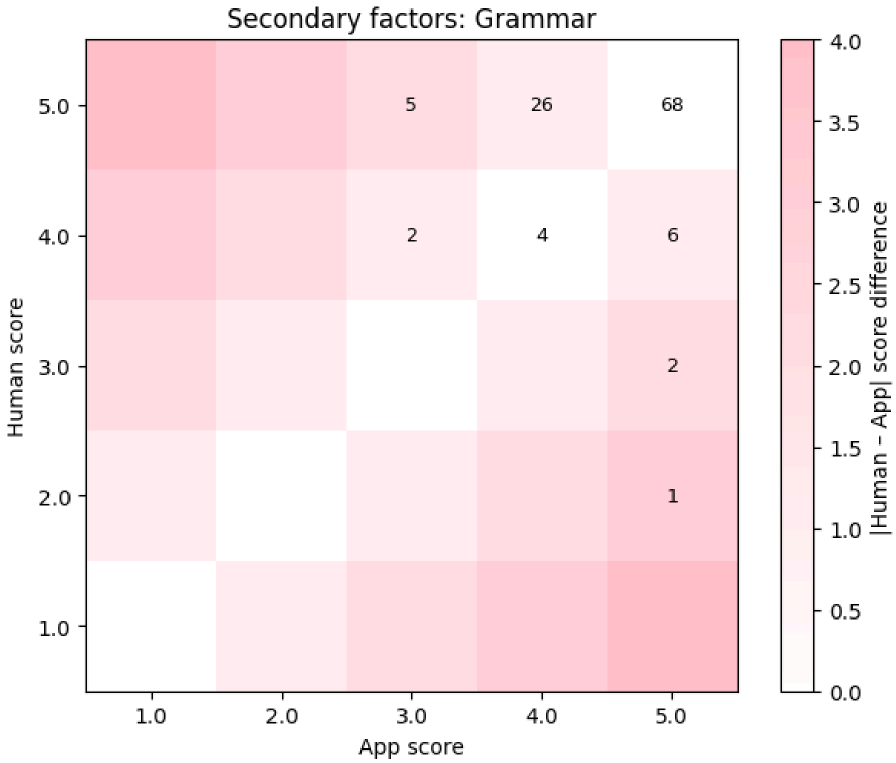
Secondary factors: Grammar
5
26
68
2
4
6
2
1
5.0
4.0
3.0
2.0
1.0
Human score
1.0
2.0
3.0
4.0
5.0
App score
4.0
3.5
3.0
2.5
2.0
1.5
1.0
0.5
0.0
|Human – App| score difference

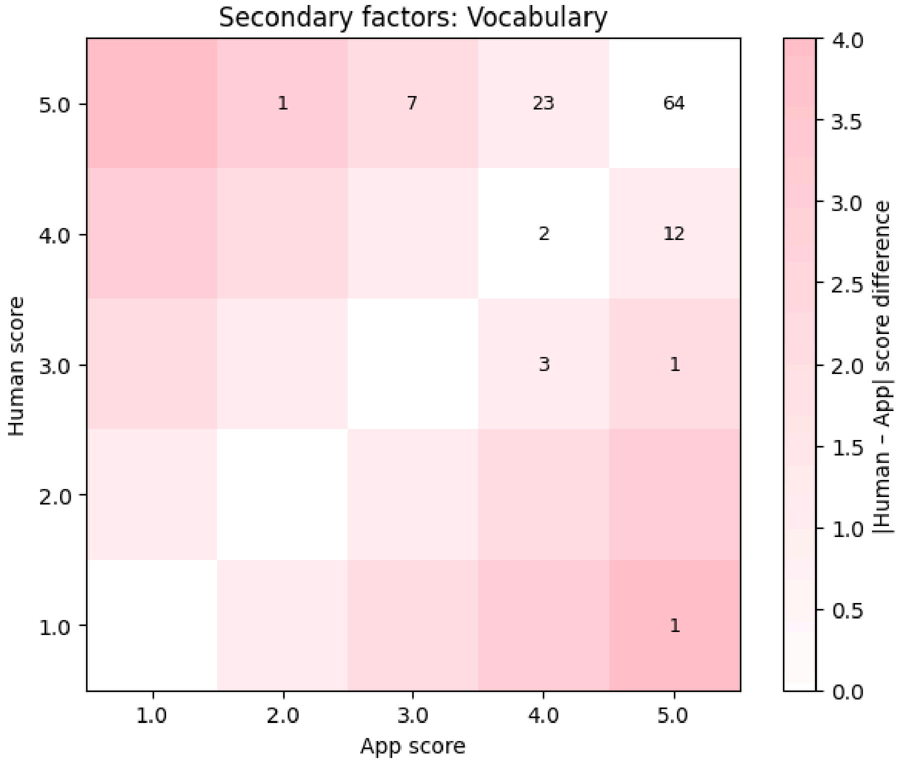
Secondary factors: Vocabulary
1
7
23
64
2
12
3
1
1
5.0
4.0
3.0
2.0
1.0
Human score
1.0
2.0
3.0
4.0
5.0
App score
4.0
3.5
3.0
2.5
2.0
1.5
1.0
0.5
0.0
|Human – App| score difference

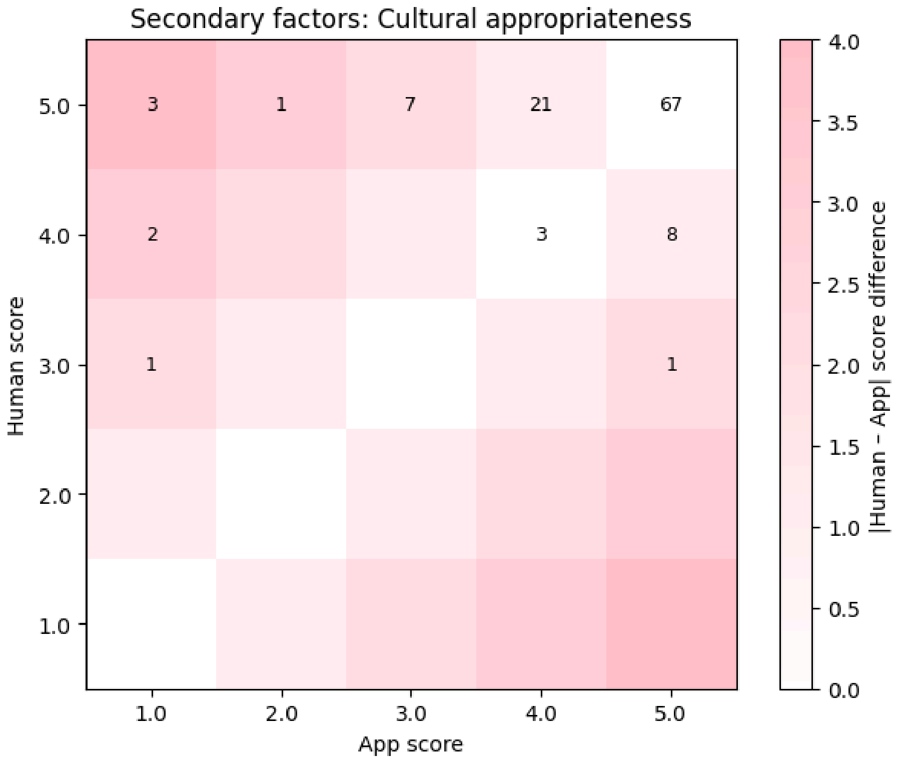
Secondary factors: Cultural appropriateness
Human score
App score
|Human – App| score difference
3
1
7
21
67
2
3
8
1
1

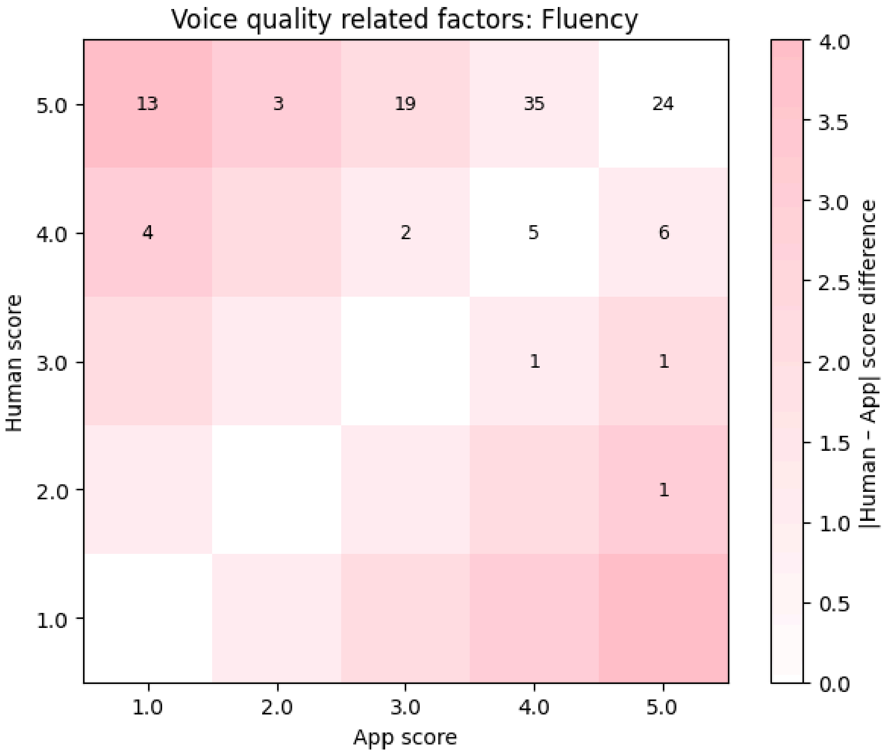
Voice quality related factors: Fluency
Human score
App score
|Human – App| score difference
13
3
19
35
24
4
2
5
6
1
1
1

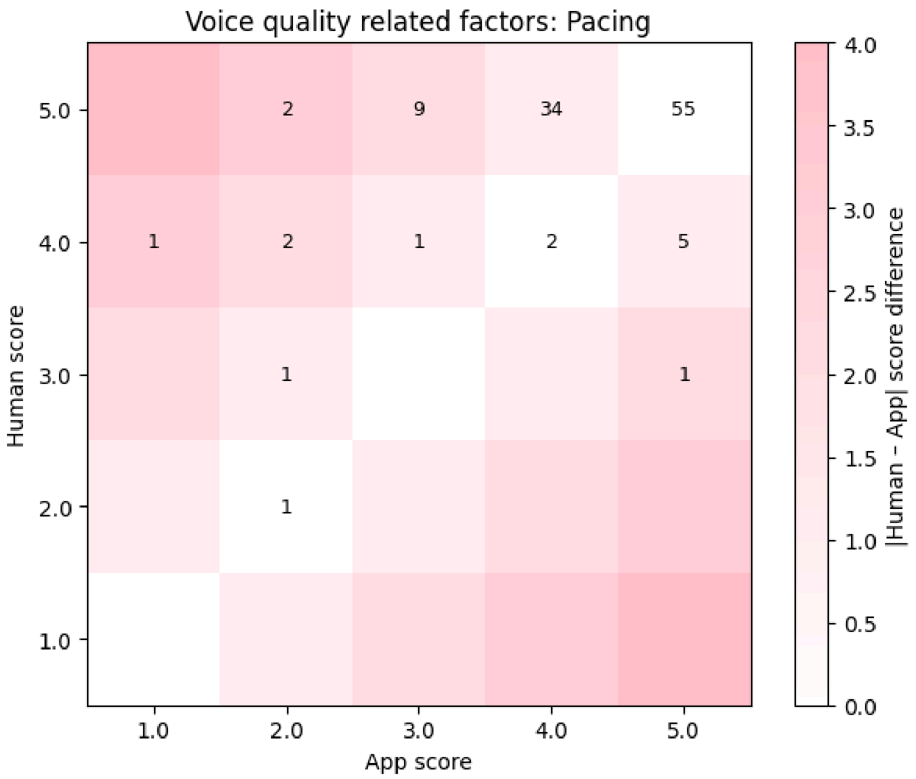
Voice quality related factors: Pacing
Human score
App score
|Human – App| score difference
5.0
4.0
3.0
2.0
1.0
2
9
34
55
1
2
1
2
5
1
1
1
0.0
0.5
1.0
1.5
2.0
2.5
3.0
3.5
4.0

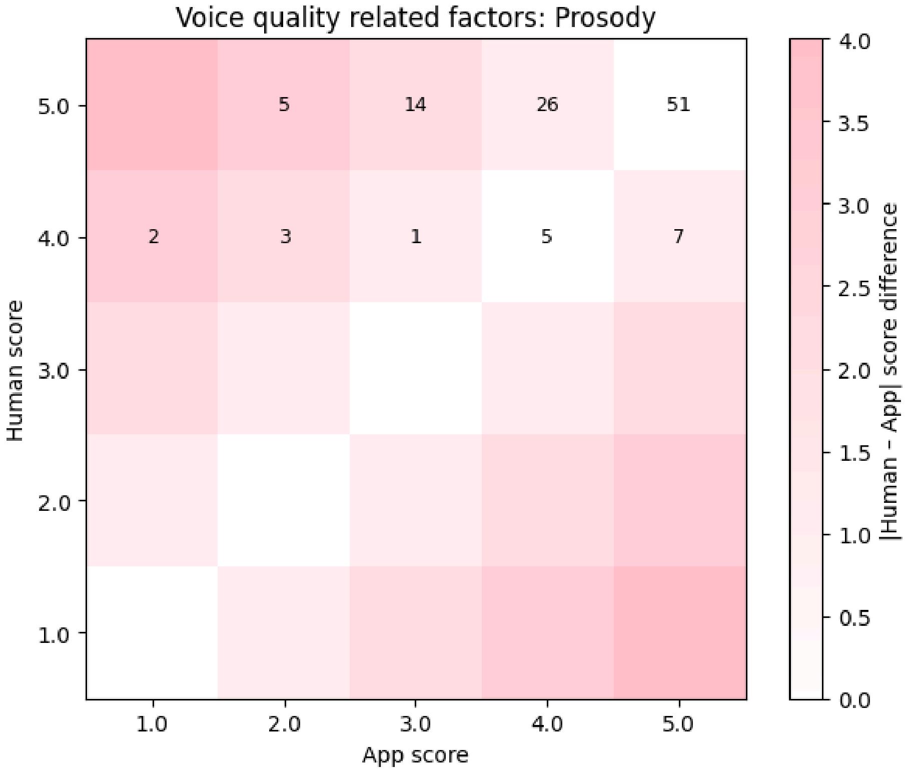
Voice quality related factors: Prosody
Human score
App score
|Human – App| score difference
5.0
4.0
3.0
2.0
1.0
5
14
26
51
2
3
1
5
7
0.0
0.5
1.0
1.5
2.0
2.5
3.0
3.5
4.0

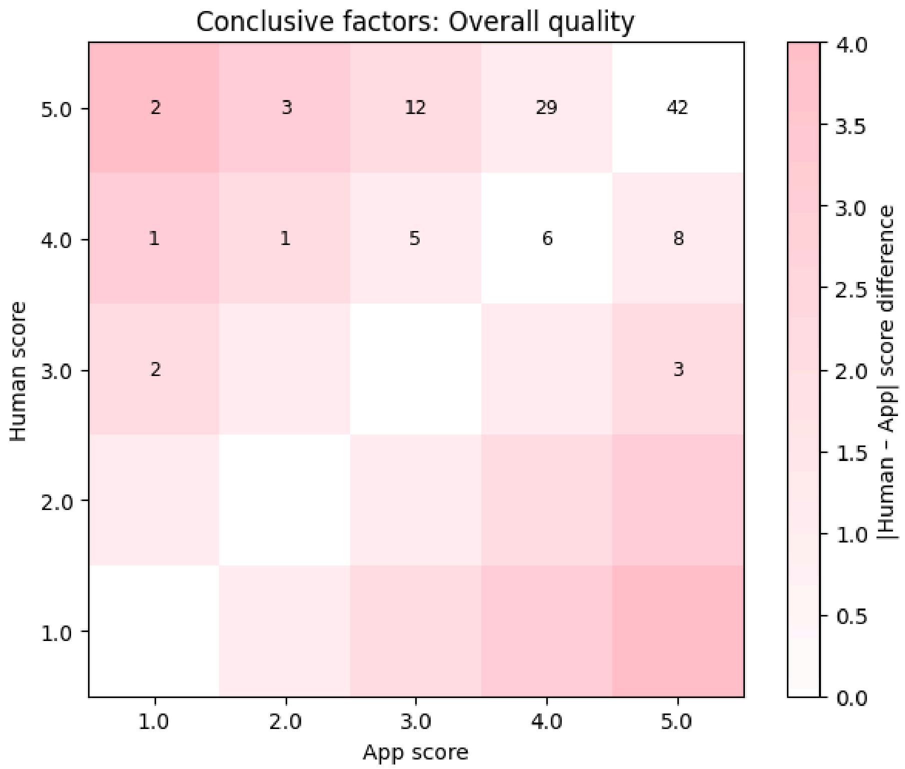

Conclusive factors: Overall quality
Human score
App score
|Human – App| score difference
5.0
4.0
3.0
2.0
1.0
2 3 12 29 42
1 1 5 6 8
2 3
0.0 0.5 1.0 1.5 2.0 2.5 3.0 3.5 4.0

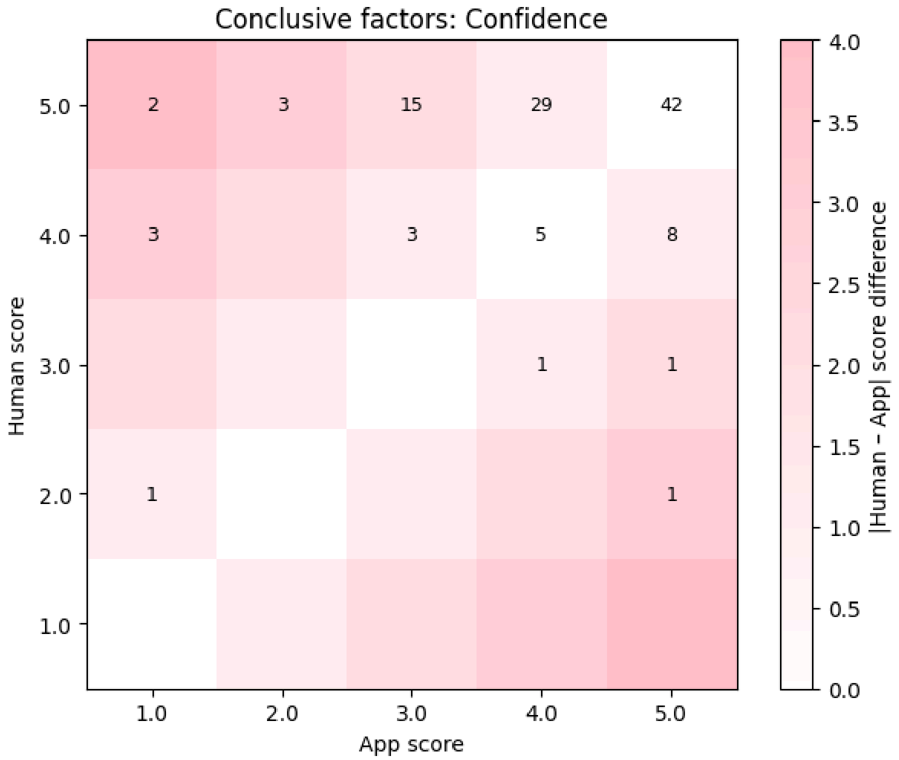

Conclusive factors: Confidence
Human score
App score
|Human – App| score difference
5.0
4.0
3.0
2.0
1.0
2 3 15 29 42
3 3 5 8
1 1
1 1
0.0 0.5 1.0 1.5 2.0 2.5 3.0 3.5 4.0

SUPPLEMENTARY MATERIAL 7 (SM7)

Error Analysis

Scenario 1:

- P2 Doctor:
  - Audio: https://drive.google.com/file/d/1mYwU7woeLh2gtEjdeKULzEnv-H6Fgsvc/view
  - Transcription: “That’s expected. The swelling should go down over the next few weeks. Are you still doing the Budesonide irrigations?”
  - Translation: Eso es normal; la hinchazón debería disminuir en las próximas semanas. ¿Todavía estás haciendo las irrigaciones de budesonida?
  - Errors: fluency/prosody
- P6 Doctor:
  - https://drive.google.com/file/d/1rVKtMuqIX6AcbgqYiNLKZV4N9HrzcUXm/view
  - Transcription: “We’ll check on you again in six weeks. If anything worsens before then— like severe pain, bleeding, or fever, call us right away.”
  - Translation: “Te revisaremos nuevamente en seis semanas. Si algo empeora antes de eso, como dolor severo, sangrado o fiebre, llámanos de inmediato.”
  - Errors: fluency/prosody

Scenario 2:

- P2 Doctor:
  - https://drive.google.com/file/d/1mJQVjuWFztOxaT55Cv_DNuubmcJnvSoC/view
  - Transcription: “Let’s go over them. You’re on Losartan for blood pressure, Metformin for diabetes, and Budesonide for your nose. Are you taking all of them as directed?”
  - Translation: “Vamos a revisarlos. Estás tomando losartán para la presión arterial, metformina para la diabetes y budesonida para la nariz. ¿Estás tomando todos ellos como se te indicó?”
  - Errors: fluency/prosody
- P5 Patient:
  - https://drive.google.com/file/d/14zvm37OCqJchT_AHIOM6ywxiGdr_L_Nw/view
  - Transcription: “Está bien. Además, me siento con mucha sed todo el tiempo.”
  - Translation: “It's okay; besides, I feel very thirsty all the time.”
  - Errors: fluency/prosody

Scenario 3:

- P1 Doctor:
  - https://drive.google.com/file/d/1Ynpm4kQbZlIQ_QpeGpzUrZWq4GQRjP8z/view
  - Transcription: "What brought you to the ER today?”
  - Translation: “¿Qué lo trajo a la sala de emergencias hoy?”

    - Errors: fluency/prosody
- P1 Patient:
    - https://drive.google.com/file/d/1NLVgqtNytZt1nfDrzNGHLMMkEYGriTyN/view
    - Transcription: “Esta mañana empecé a sentir dolor en el pecho. Se siente como una fuerte presión.”
    - Translation: “This morning I started to feel pain in my chest; it feels like a strong pressure.”
    - Errors: fluency/prosody

SUPPLEMENTARY MATERIAL 8 (SM8)

Translation Latency and Estimated cost per 10-minute conversation

Based on internal analytics from the LingualAI system, the average end-to-end translation latency—including speech-to-text (STT), real-time partial translation, transcription verification and correction, and final sentence-level translation is 9.72 seconds per message. This reflects the average duration from the start of a spoken sentence to the playback of its translated audio (text-to-speech output) in the target language. The latency is sufficiently low to support effective turn-based conversations in clinical settings.

Cost Estimate for a 10-Minute Medical Conversation

To contextualize the economic impact, we compared the estimated cost of a 10-minute bilingual clinical conversation using LingualAI against conventional interpreter services.

1. LingualAI (AI-based pipeline)

- Average words per message: 14.45
- Messages per 10-minute session (based on 9.72s average duration): approximately 61.7 messages
- Total words per session: 14.45 × 61.7 ≈ 891 words

Translation API usage per 10-minute session (OpenAI GPT-4o-mini)

- Real-time partial translations: ~154 calls (Assuming an average of 2.5 translation calls per message during STT partial updates)
- Final sentence-level translation: 61.7 calls
- STT verification and correction (post-STT): 61.7 calls
- Total estimated OpenAI API calls: 277.4 (rounded to 278 requests)

Cost Calculation (GPT-4o-mini model)

- OpenAI pricing: $0.15 per 1 million input tokens
- Estimated tokens per translation call: 25 tokens (based on ~18–22 words per message)
- Total input tokens per session: 278 × 25 = 6,950 tokens
- Translation cost: (6,950 ÷ 1,000,000) × $0.15 ≈ $0.0010425 USD

Infrastructure Cost (AWS Services)
Based on AWS billing data for S3, Lambda, API Gateway, DynamoDB, and Cognito used through AWS Amplify:

- Total AWS cost for 3,456 sessions ≈ $92 USD
- Average cost per session ≈ $0.027 USD
- Rounded estimate for infrastructure cost per 10-minute conversation ≈ $0.03 USD

Total Estimated Cost Using LingualAI

- Translation API (OpenAI): ≈ $0.001

- Infrastructure (AWS): ≈ $0.03
- Total: approximately $0.03–$0.04 USD per 10-minute conversation

2. Human Interpreter (Phone / Video Interpretation)

Based on enterprise invoices from June 2025:

- Phone Interpretation (Language Line Services):
- 508 minutes → $350.52 → $0.69 per minute
- Video Interpretation (Insight):
- 1,221 minutes → $1,288.76 → $1.06 per minute

Estimated Interpreter Cost per 10-Minute Session

- Phone Interpreter: $6.90
- Video Interpreter: $10.60

Conclusion: Practical Trade-Off

LingualAI achieves sub-10-second translation latency per sentence, enabling efficient turn-based communication in clinical settings. Compared to traditional phone or video interpretation services, the cost of a 10-minute medical conversation is reduced from $6.90–$10.60 to just $0.03–$0.04, a greater than 99.5% reduction. This demonstrates the scalability and cost-effectiveness of AI-driven translation, especially for high-demand or resource-limited healthcare environments.
